# The mechanism of PKM2/HIF-1α axis polarizing TAMs by upregulating glucose-serine metabolism to promote melanoma progression

**DOI:** 10.3389/fimmu.2026.1740155

**Published:** 2026-03-31

**Authors:** Ying Yang, Peng Su, Xinqi Yang, Na Liang, Xiang Huang, Ying Sun, Changxian Chen, Hua Chen, Chunming Li, Jiawei Yang, Neng Zhang

**Affiliations:** 1 Department of Dermatology, The Second Affiliated Hospital of Zunyi Medical University, Zunyi, Guizhou, China; 2 Department of Pathology, Affiliated Hospital of Zunyi Medical University, Zunyi, Guizhou, China; 3 Department of Urology, Affiliated Hospital of Zunyi Medical University, Zunyi, Guizhou, China; 4 Department of Dermatology, Jiangsu Provincial People’s Hospital Chongqing Hospital, Chongqing, China; 5 Department of Biochemistry, Zunyi Medical University, Zunyi, Guizhou, China

**Keywords:** *de novo* serine synthesis, glucose-serine metabolism, HIF-1α, melanoma, PKM2, tumor-associated macrophages(TAMs)

## Abstract

**Background:**

Melanoma is the most aggressive skin cancer. Tumor-associated macrophages (TAMs) promote melanoma progression through polarization, however, whether glucose-serine metabolism regulates TAMs polarization and the specific regulatory mechanism remain unclear.

**Methods:**

Immunohistochemistry (IHC) was used to detect the infiltration of M2-type TAMs in melanoma tumor tissues; bioinformatics analysis was employed to identify HIF-1α, a key gene regulating glucose metabolism and polarization of TAMs; *in vivo* and *in vitro* experiments were conducted to determine whether HIF-1α promotes melanoma progression by regulating TAMs polarization and to explore the regulatory mechanism of HIF-1α on glucose-serine metabolism and polarization in melanoma TAMs; Co-IP, immunofluorescence, and functional rescue experiments were used to verify the upstream regulatory factors of HIF-1α.

**Results:**

M2-type TAMs were highly enriched in melanoma tumor tissues and closely associated with tumor progression and poor prognosis. Single-cell sequencing data of melanoma suggested that HIF-1α is a key gene regulating glucose metabolism and polarization in TAMs. *In vivo* and *in vitro* experimental results demonstrated that HIF-1α upregulated glucose–serine metabolism in melanoma TAMs, thereby driving their polarization toward the M2 phenotype and consequently promoting tumor progression. PKM2 acted as an upstream regulatory factor of HIF-1α, and the PKM2/HIF-1α axis upregulated glucose-serine metabolism in TAMs to induce polarization. Additionally, a self-reinforcing circuit regulatory relationship among PKM2/HIF-1α/serine was identified within melanoma TAMs.

**Conclusions:**

Our study reveals that the PKM2/HIF-1α axis upregulates glucose–serine metabolism to induce M2 polarization of TAMs and drive melanoma progression. Targeting this metabolic axis thus holds promise as a novel therapeutic strategy for melanoma patients.

## Introduction

Melanoma is the most malignant type of skin tumor. Its complex pathogenesis and the immunosuppressive tumor microenvironment (TME) contribute to its highly metastatic, rapidly progressive, and highly malignant characteristics, resulting in poor prognosis and high mortality in patients (1–3). As the most abundant infiltrating immune cells within the TME, tumor-associated macrophages (TAMs) act as pivotal regulators by polarizing into distinct phenotypes, thereby facilitating tumor progression through multiple mechanisms (4, 5). Consequently, an in-depth investigation into the regulatory mechanisms of TAMs polarization in melanoma is crucial for inhibiting melanoma progression.

Metabolic reprogramming refers to a process by which cells alter their metabolic pathways to adapt to survival and functional demands under specific environments (6).

Studies have demonstrated that metabolic reprogramming can induce TAMs polarization through epigenetic modifications or metabolite-driven regulation of key transcription factors (7, 8). However, whether metabolic reprogramming in melanoma TAMs can induce polarization and the underlying mechanisms remain elusive. Metabolic reprogramming of TAMs primarily occurs in three aspects: glucose, amino acids, and lipids. As the principal energy source for TAMs, glucose metabolism is intimately linked to macrophage polarization status. Traditional studies have established that M1-type macrophages predominantly rely on glycolysis for energy acquisition, whereas M2-type macrophages depend on tricarboxylic acid (TCA) cycle-driven oxidative phosphorylation (OXPHOS) for energy supply (9–11). However, emerging evidence suggests that glycolysis also contributes to M2-type TAMs polarization. For instance, glycolysis-related proteins are upregulated in lung cancer TAMs (12), and the glycolytic inhibitor 2-deoxy-D-glucose (2-DG) can suppress M2 polarization through the AMPK signaling pathway, thereby attenuating tumor immune evasion (13).The *de novo* serine synthesis pathway (SSP), a critical branch of glycolysis (14), has been implicated in macrophage polarization. Phosphoglycerate dehydrogenase (PHGDH) serves as the key rate-limiting enzyme of the SSP. Jayne et al. demonstrated that PHGDH overexpression promotes IL-4-induced macrophage polarization toward the M2-type (15). Additionally, Xiao Shan et al. revealed that serine facilitates macrophage M2 polarization through the IGF1-p38 axis (16). yet relevant research in TAMs remains limited. Given the crucial regulatory roles of glucose metabolism and SSP in macrophage polarization, our study aims to investigate whether glucose-serine metabolic reprogramming can induce melanoma TAMs polarization and elucidate the underlying regulatory mechanisms.

HIF-1α, a key transcription factor in hypoxic environments, participates in glucose metabolism regulation through multiple signaling pathways. However, whether HIF-1α can induce the polarization of melanoma TAMs by modulating intracellular glucose-serine metabolism and the specific underlying mechanisms remain elusive. Current studies indicate that HIF-1α can activate the transcription of glucose transporters and glycolysis-related genes; however, research on its regulation of serine metabolism remains limited. Only a few studies have suggested that HIF-1α may modulate amino acid metabolism, such as α-ketoglutarate and arginine, in TAMs (17, 18). Pyruvate kinase M2 (PKM2) is a key glycolytic enzyme and exhibits a mutual regulatory relationship with HIF-1α: on one hand, HIF-1α can activate PKM2 transcription (19, 20); on the other hand, PKM2 also promotes HIF-1α expression (21). For instance, TIPE enhances glycolytic capacity in tumor cells through the PKM2/HIF-1α axis (22). Whether the PKM2/HIF-1α axis can induce melanoma TAMs polarization through regulating glucose-serine metabolism requires in-depth investigation. Elucidation of this mechanism may provide potential therapeutic targets for inhibiting tumor progression by suppressing melanoma TAMs polarization.

In this study, we found that melanoma cells induce M2 polarization of TAMs, which is closely associated with poor prognosis in melanoma patients. Through bioinformatics analysis, we identified HIF-1α as a key gene regulating melanoma TAMs polarization. Subsequent *in vitro* and *in vivo* experiments demonstrated that HIF-1α promotes M2 polarization of melanoma TAMs by upregulating glucose-serine metabolism, thereby accelerating tumor progression. Furthermore, co-immunoprecipitation (Co-IP), immunofluorescence (IF), and functional rescue experiments confirmed that PKM2 acts as an upstream regulator of HIF-1α. In summary, our study reveals that melanoma induces M2 polarization of TAMs, and that the PKM2/HIF-1α axis promotes tumorigenic effects by upregulating glucose-serine metabolism in melanoma TAMs, providing a theoretical basis and potential therapeutic targets for inhibiting tumor progression by targeting glucose-serine metabolism. Additionally, our study identified a self-reinforcing circuit among PKM2, HIF-1α, and serine in melanoma TAMs.

## Materials and methods

### Clinical specimens

Tumor tissues and adjacent normal tissues were retrospectively collected from melanoma patients who underwent surgical resection without prior treatment at the Affiliated Hospital of Zunyi Medical University between January 2019 and May 2024. The inclusion criteria were as follows: (1) histopathological diagnosis of melanoma confirmed independently by at least two pathologists according to the American Joint Committee on Cancer (AJCC) guidelines; (2) All patients underwent radical surgical resection, ensuring complete and intact specimen retrieval for subsequent analysis; (3) melanoma located at acral sites as well as non-acral sites, including trunk and extremities; (4) complete clinical and pathological data available; and (5) age ≥18 years. The exclusion criteria were: (1) presence of other concurrent malignant tumors; (2) history of preoperative radiotherapy, chemotherapy, or immunotherapy; (3) comorbid autoimmune diseases; and (4) mucosal or uveal melanoma.

A total of 65 melanoma patients were enrolled, comprising 46 cases of acral melanoma and 19 cases of cutaneous melanoma. According to the AJCC 8th edition staging system, the cohort included 7 patients with stage I, 19 with stage II, 15 with stage III, and 24 with stage IV disease. None of the patients received antitumor therapy prior to sample collection. All patients were followed up postoperatively until December 2024, with a minimum follow-up duration of 6 months. This study was approved by the Ethics Committee of the Affiliated Hospital of Zunyi Medical University (approval number: KLLY-2023-231).

### Immunohistochemistry and immunohistochemical analysis

The excised tissues were fixed in 10% formalin solution, subjected to gradient dehydration, embedded in paraffin wax, and subsequently sectioned into 3-μm-thick serial sections. For HE staining, the sections were first stained with hematoxylin solution. Subsequently, excess hematoxylin was removed using 1% hydrochloric acid alcohol, followed by staining with eosin solution. After gradient dehydration, the sections were mounted with neutral balsam for sealing.

Immunohistochemistry (IHC) staining. Following antigen retrieval, the sections were incubated overnight at 4 °C with CD68 antibody (1:1000, 84596-4-RR, Proteintech) and CD163 antibody (1:5000, 68218-1-Ig, Proteintech) respectively. After washing, sections were incubated with secondary antibodies and visualized using DAB. Finally, the sections were counterstained with Mayer’s hematoxylin.

Immunohistochemical scoring criteria. Five representative fields with TAMs infiltration were initially selected at low magnification (×100). Staining intensity and the percentage of positive macrophages were subsequently assessed at high magnification (×400) using a semi-quantitative scoring system: Staining intensity score: 0 = no visible staining; 1 = light yellow; 2 = moderate yellow; 3 = deep yellow; 4 = brown. Staining proportion score: 0 = no positive cells; 1 = 1%-25% positive cells; 2 = 26%-50% positive cells; 3 = 51%-75% positive cells; 4 = ≥76% positive cells. The total score (intensity score × proportion score) was used for stratification: negative (score 0), low expression (score ≤4), or high expression (score >4).

### Download of melanoma scRNA-seq data and KEGG enrichment analysis

Single-cell sequencing(scRNA-seq) data of two acral melanoma samples (GSE189889 and GSE215120) were downloaded from the GEO database (https://www.ncbi.nlm.nih.gov/geo/) and analyzed using Python-based toolkits, including Scanpy v1.11.5 and clusterProfiler v4.6.0. After quality control filtering of the raw count matrix, data normalization was performed via log1p transformation and scaling, followed by selection of the top 2000 highly variable genes (HVGs). Batch effects were eliminated using scVI v1.4.1. Dimensionality reduction was conducted with Uniform Manifold Approximation and Projection (UMAP), and cell clustering was achieved by the Leiden algorithm with a resolution of 0.8. M1 and M2 TAMs were identified using the specific markers CD86/TNF-α/IL-12B/IL-23A and CD206/IL-10/CD163, respectively. Differentially expressed genes (DEGs) between the two groups were screened with the rank_genes_groups function in the Scanpy package under the thresholds of |log2 fold change (log2FC)| > 1 and adjusted *P* value (padj) < 0.05 to construct the DEG dataset. The intersection of this dataset with the glucose metabolism gene set retrieved from the Molecular Signatures Database (MSigDB) was obtained, and Kyoto Encyclopedia of Genes and Genomes (KEGG) enrichment analysis was subsequently performed using the clusterProfiler package with the cutoffs of *P* < 0.05 and q < 0.05. Finally, the key signaling pathways and core driver genes regulating TAMs polarization were identified from the enrichment results.

### Cell culture and transfection

This study used two melanoma cell lines, A375 and SK-MEL-28, as well as the human acute monocytic leukemia cell line THP-1. All three cell lines were obtained from the Cell Bank of the Chinese Academy of Sciences (Shanghai, China). All cell lines were routinely confirmed to be negative for mycoplasma. A375 and SK-MEL-28 cells were cultured in DMEM medium (Pricella, Wuhan, China), while THP-1 cells were cultured in RPMI-1640 medium (Pricella, Wuhan, China). All cell culture media were supplemented with 10% fetal bovine serum (Gibco, NY, USA) and 1% penicillin/streptomycin (Invitrogen, CA, USA). All lines were maintained in a humidified incubator at 37 °C with 5% CO_2_.

Lentiviral vectors targeting HIF-1α were constructed by Shanghai Genechem Co. Ltd. (Shanghai, China). THP-1 cells were infected with lentiviral particles and selected in 2 μg/mL puromycin (ECOTOP, Guangzhou, China) for 10 days to establish stable HIF-1α-knockdown cell lines. Successful HIF-1α knockdown was verified by Western blot. Control cells were infected with empty vectors, and all transduction and selection procedures were performed identically to the knockdown group.

### Cell treatment

To obtain M0 macrophages, 5 mL of THP-1 cells in the logarithmic growth condition were centrifuged at 800 rpm for 5 min to harvest cell pellets. The pellets were resuspended in complete medium supplemented with 100 ng/mL PMA (MCE, USA) by pipetting. Subsequently, the THP-1 cell suspension was transferred into T25 cell culture flasks. After 24 h of incubation, the successful differentiation of THP-1 cells into M0 macrophages was confirmed under a microscope when the suspended cells switched to an adherent phenotype. The cell conditioned medium of A375 and SK-MEL-28 was collected separately, centrifuged at 3000 rpm for 10 minutes, and then filtered through a 0.22 μm filter. The filtered cell conditioned medium was mixed with serine-free complete medium (DM150110, Pricella, Wuhan, China) at a 1:1 ratio to prepare “co-culture mixtures”. M0 macrophages were treated with the “co-culture mixtures” for 48 hours, which induced M0 macrophages to differentiate into A375-TAMs and SK-TAMs, respectively. A375-TAMs with HIF-1α gene knockdown were named A-TAMs-si, and SK-TAMs with HIF-1α gene knockdown were called S-TAMs-si.

To investigate the mechanism underlying the polarization of TAMs, different treatments were administered during TAMs induction, including glycolysis inhibition, serine metabolism inhibition, PKM2 gene activation, and exogenous serine supplementation. Based on the drug concentrations used in other relevant studies, the specific treatments and selected drug concentrations for each group are as follows: 1) TAMs+2-DG: M0 macrophages were pretreated with 1000 μM 2-DG, a glycolysis inhibitor, for 3 h, followed by incubation with the “co-culture mixtures” for an additional 48 h to generate glycolysis-inhibited TAMs (23). 2) TAMs+NCT503: M0 macrophages were pretreated with 10 μM NCT503, a serine metabolism inhibitor, for 3 h prior to a 48-h incubation with the “co-culture mixtures”, to generate serine metabolism-inhibited TAMs (24). 3) TAMs+TEPP-46: M0 macrophages were pretreated with 500 nM TEPP-46 (25), a PKM2 activator, for 12 h, and then cultured with the “co-culture mixtures” for 48 h to obtain PKM2-activated TAMs. 4) TAMs+L-ser: M0 macrophages were directly incubated with the “co-culture mixtures” supplemented with 100 μmol/L exogenous serine for 48 h to generate serine-supplemented TAMs (26).

To investigate the effects of TAMs on the malignant biological behaviors of tumor cells, the cell culture supernatants of M0 macrophages, TAMs, and TAMs-si groups were collected, centrifuged at 3000 rpm for 10 min, and then filtered through a 0.22 μm filter membrane. The filtered conditioned media from M0/TAMs/TAMs-si cells were subsequently used to treat A375 and SK-MEL-28 cells for wound healing assay, migration assay, and invasion assay. In addition, the filtered conditioned media from M0/TAMs/TAMs-si cells were mixed with complete medium at a 1:1 ratio, and the mixture was used to treat A375 and SK-MEL-28 cells for colony formation assay and CCK-8 assay.

### mRNA extraction and qPCR analyses

Total RNA was extracted separately from nude mouse subcutaneous xenograft tumor tissues and TAMs induced with melanoma cell supernatants using RNAkey reagent (SEVEN, Beijing, China). Complementary DNA (cDNA) was synthesized with the PrimeScript™ RT reagent Kit (Takara, Dalian, China). For mRNA expression analysis, quantitative real-time polymerase chain reaction (qRT-PCR) was performed using TB Green^®^ Premix Ex Taq™ II reagent on the CFX Connect™ quantitative PCR detection system (BIO-RAD, USA). The PCR reaction conditions were as follows: 94 °C for 30 s, followed by 40 cycles of 94 °C for 15 s, 61 °C for 35 s, and 72 °C for 25 s; followed by 95 °C for 10 s, 65 °C for 60 s, and 97 °C for 1 s. Homo sapiens b-actin was used as internal standard control. Relative mRNA expression levels of the gene of interest were calculated using the 2^-△△Ct^ method. All primer sequences used were shown in the **Supplementary Table 1**.

### Western blot

Nude mouse subcutaneous xenograft tumor tissues and TAMs were lysed using RIPA buffer (Beyotime, Shanghai, China) supplemented with protease inhibitors (Beyotime, shanghai, China). The extracted proteins were quantified using a BCA protein concentration assay kit (Solarbio, Beijing, China). Equal amounts of proteins were separated by sodium dodecyl sulfate-polyacrylamide gel electrophoresis (SDS-PAGE), followed by Western blotting to analyze the expression levels of target proteins using specific antibodies. After blocking the blots with a protein-free rapid blocking solution, they were incubated overnight at 4 °C with primary antibodies targeting respective target proteins. The next day, the membranes were incubated with the HRP-conjugated secondary antibodies at room temperature for 1 h. Finally, protein expression levels were detected using an automatic chemiluminescence image processing system (Bio-Rad, CA, USA). The antibodies used in this study are listed in **Supplementary Table 2**.

### Flow cytometry

TAMs induced with melanoma-conditioned medium were trypsinized and resuspended in phosphate-buffered saline (PBS) at a concentration of approximately 1×10^6^ cells/mL. After blocking, the cells were incubated separately with CD163 antibody (556018, BD) and CD206 antibody (321106, BioLegend). Subsequently, flow cytometry analysis (FACS) was performed using a Facscalibur flow cytometer (BD, New Jersey, USA).

### Immunofluorescence

For the cellular immunofluorescence assay, A375-TAMs and SK-MEL-28-TAMs were generated in 48-well plates. After washing twice with PBS, the cells were fixed with 4% paraformaldehyde (Solarbio, Beijing, China), followed by permeabilization with 0.2% Triton X-100 (Solarbio, Beijing, China). After blocking with non-fat milk, the cells were incubated with anti-HIF-1α antibody (1:400, 20960-1-AP, Proteintech) for 1 h. Subsequently, a Cy3-conjugated goat anti-rabbit IgG fluorescent secondary antibody (A10520, Thermo Fisher Scientific) was added and incubation. Finally, the cell nuclei were counterstained with DAPI (Beyotime, Shanghai, China). After mounting, the cells were observed under a fluorescence microscope (Nikon, Tokyo, Japan).

For immunofluorescence staining of tissue sections, the sections were first dewaxed, followed by antigen retrieval. After blocking with serum, HIF-1α antibody (1:400, 20960-1-AP, Proteintech) was added and incubated overnight at 4 °C. After re-blocking with serum, the CD163 antibody (1:400, 68218-1-Ig, Proteintech) was added and incubated overnight at 4 °C. Finally, cell nuclei were counterstained with DAPI (Beyotime, Shanghai, China), and the sections were observed under a fluorescence microscope (Nikon, Tokyo, Japan). The Mean Fluorescence Intensity (MFI) was quantified from confocal laser scanning microscopy images using ImageJ software. Regions of interest (ROIs) were manually drawn along the cell boundary based on the corresponding DAPI staining. MFI was calculated as integrated density divided by ROI area.

### Cell counting kit-8 assay

Melanoma cells were seeded in 96-well plates at a density of 3000 cells per well, and divided into three groups. The three groups were treated respectively with mixed solutions consisting of M0, TAMs, or TAMs-si cell culture supernatants mixed with complete medium at a 1:1 ratio.

Referring to the kit instructions, the CCK-8 assay (ECOTOP, Guangzhou, China) was used to detect the optical density (OD) value at 450 nm of cells in each group after 24 h, 48 h, and 72 h of culture, and cell viability was calculated. Cell viability was calculated using the formula. Cell Viability(%) = (OD value of experimental group - OD value of blank group)/(OD value of control group - OD value of blank group) × 100%. This experiment was conducted to investigate the effect of TAMs on tumor cell viability.

M0 macrophages were seeded in 96-well plates at a density of 6000 cells per well, and treated with “co-culture mixtures” containing different concentrations of 2-DG or NCT503 for 48 hours. For 2-DG, four concentrations were set: 0, 500, 1000, and 2000 μM. For NCT503, four concentrations were set: 0, 5, 10, and 20 μM. After 48 hours of incubation, the OD values of TAMs in each group were measured at 450 nm using a CCK-8 kit, and cell viability was calculated.

### Wound healing assay

Melanoma cells in logarithmic growth phase were seeded in 6-well plates and cultured in DMEM containing 10% FBS until the cell density reached approximately 90%. Scratches were created using a 200 μL pipette tip on the cell monolayer. After washing with PBS, the scratched areas were imaged using a microscope (Olympus, Tokyo, Japan). The cells were divided into three groups, and cell culture supernatants from M0/TAMs/TAMs-si were added to each group, respectively, for further culture. After 24 h, the healed areas were photographed again using a microscope (Olympus, Tokyo, Japan). The scratch healing rate of melanoma cells in each group was analyzed using Image Pro Plus software.

### Invasion and migration

8.0μm Transwell chambers (Corning, NY, USA) were used to measure cell invasion and migration ability. For the migration assay, the upper chamber of the transwell was used directly; for the invasion assay, Matrigel (Corning, NY, USA) needed to be pre-added inside the upper chamber of the Transwell. Melanoma cells in logarithmic growth phase were trypsinized and centrifuged. The cell pellets were divided into three groups, resuspended in M0, TAMs, or TAMs-si cell culture supernatants, respectively, and adjusted to a concentration of 5×10^5^ cells/mL. 100 μL of the cell suspension from each group was seeded into the upper chamber of the transwell chamber. Subsequently, 600 μL of RPMI 1640 medium containing 10% FBS was added into the lower chamber. After 24 h of incubation in a cell culture incubator, the cells that had migrated to the lower surface of the membrane was fixed with 4% paraformaldehyde and stained with 0.1% crystal violet. The average number of invading/migrating cells was quantified from six randomly selected microscopic fields.

### Co-IP

Total protein was extracted from TAMs and used for Co-IP. An appropriate volume of primary antibody was added to the protein lysate, and the mixture was gently shaken overnight at 4 °C to promote the formation of antigen-antibody complexes. Subsequently, Protein A+G agarose magnetic beads were added to the lysate, followed by further incubation at 4 °C for 2 h. Magnetic beads bound to protein complexes were collected using a magnetic stand and thoroughly washed five times with lysis buffer. Subsequently, the protein complexes were resuspended in SDS loading buffer, and immunocomplexes were analyzed by Western blotting.

### Amino acid metabolomics analysis

TAMs and TAMs-si were first digested with trypsin and then centrifuged to collect cells. The cell pellets were sent to Wuhan LingSi Biotechnology Co., Ltd. (Wuhan, China) for amino acid metabolomics analysis.

### Xenografted tumor model

Male BALB/c nude mice (4 weeks old) were purchased from Changzhou Cavens Model Animal Co., Ltd. (Changzhou, China) and maintained under specific pathogen-free (SPF) conditions. All animal experimental protocols were approved by the Institutional Animal Care and Use Committee (IACUC) of Zunyi Medical University (Approval No.: zyfy-an-2024-0583). Experimental mice were divided into three groups using a random number table method to ensure baseline consistency among all groups. the corresponding cell suspensions were subcutaneously injected into the right dorsal side of each mouse: (1) 1×10^6^ A375 cells; (2) 1×10^6^ A375 cells mixed with an equal number of TAMs; (3) 1×10^6^ A375 cells mixed with an equal number of TAMs-si. Tumor sizes were measured every 3 days by two independent researchers using a Vernier caliper. Tumor volumes were calculated using the formula: Volume (mm³) = a×b²/2, where a and b represented the maximum and minimum tumor diameters, respectively. Mice were euthanized on the 15th day post-inoculation, or when tumor volumes reached the ethical endpoint (~1000 mm³). Tumor tissues were then excised and photographed. All procedures were performed under sterile conditions to ensure animal welfare and data reliability. Blinding was applied throughout the entire process of animal model establishment, index detection, and result analysis, Both experimental operators and result analysts were kept unaware of the animal grouping information. This effectively avoided the impact of subjective bias on the experimental results.

### Statistical analysis

SPSS 29.0 (IBM Corp, Armonk, NY, USA) was used for statistical analyses, and GraphPad Prism 5.0 (GraphPad Software, San Diego, CA, USA) was used for graphing. Categorical data were analyzed using the chi-square test. For continuous data, normality testing was performed first: Normally distributed data were compared using the independent samples *t*-test (for two groups) or one-way analysis of variance (ANOVA; for multiple groups), followed by the Bonferroni test for pairwise comparisons; non-normally distributed data and multi-group ordinal variables were analyzed using the nonparametric rank-sum test (Mann-Whitney U test). Survival analysis was conducted via Kaplan-Meier analysis. Differences were considered statistically significant when *P* < 0.05.

## Result

### High infiltration of M2-type TAMs in cutaneous melanoma tissues is associated with inferior survival outcomes in patients

**Figure 1:**
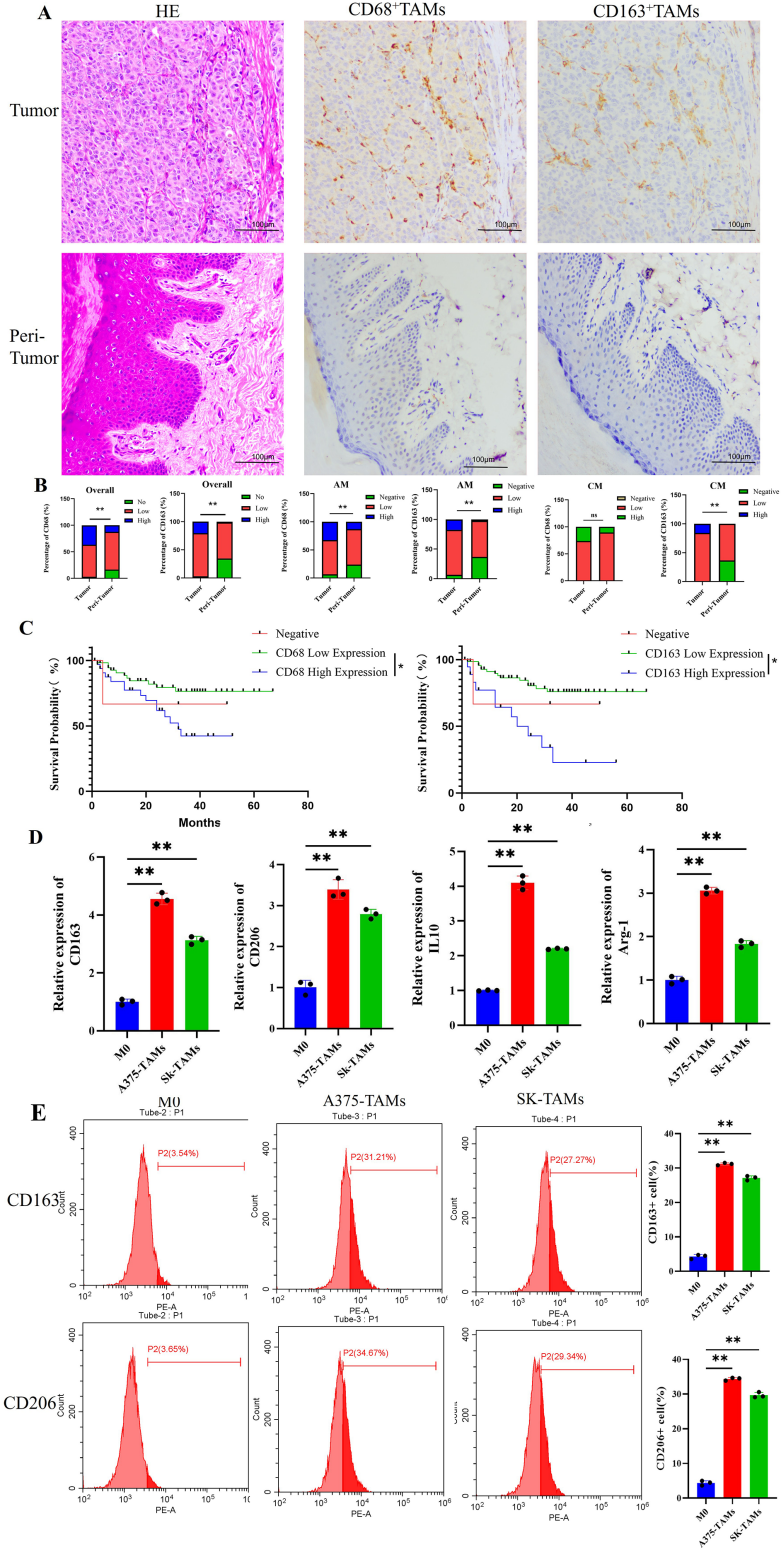
Melanoma induces the polarization of macrophages toward the M2 phenotype, and M2-type macrophages are associated with poor prognosis in melanoma patients. **(A)** HE staining and IHC staining images of CD68^+^TAMs and CD163^+^TAMs in melanoma tumor tissues and adjacent tissues (200× magnification, scale bar = 100 μm); **(B)** The infiltration levels of CD68^+^TAMs and CD163^+^TAMs in cutaneous melanoma tumor tissues were higher than those in adjacent tissues(AM: n=46,CM: n=19, and statistical analysis was performed using Mann-Whitney U test); **(C)** Melanoma patients with high infiltration of CD68^+^TAMs and CD163^+^TAMs had a poorer prognosis(statistical analysis was performed using Kaplan-Meier survival analysis); **(D)** The mRNA expression of M2-type macrophage marker genes was upregulated in TAMs induced by melanoma cell culture supernatant (n=3 per group, error bars represent SD, and statistical analysis was performed using one-way ANOVA); **(E)** The percentage of M2-type macrophages was increased in TAMs induced by melanoma cell culture supernatant (n=3 per group, error bars represent SD, and statistical analysis was performed using one-way ANOVA). *P<0.05, **P<0.01.

A total of 65 melanoma tissue samples were collected in this study. HE staining revealed that tumor cells exhibited variable morphology and size, with enlarged hyperchromatic nuclei, visible mitotic figures, and prominent nucleoli, accompanied by abundant inflammatory cell infiltration among the tumor cells. IHC staining was performed using CD68 to label total TAMs and CD163 to label M2-type TAMs. The results showed that CD68-positive expression was predominantly localized in the cytoplasm, whereas CD163-positive expression was localized in both the cytoplasm and cell membrane. CD68^+^ TAMs and CD163^+^ TAMs in tumor tissues were mainly observed as yellowish-brown or brown granular staining (**Figure 1A**).

Based on IHC staining results, statistical analysis was performed to evaluate the infiltration levels of CD68^+^ TAMs and CD163^+^ TAMs in tumor tissues and adjacent normal tissues.The results showed that CD163^+^ TAMs infiltration was significantly higher in tumor tissues than in adjacent tissues in the overall cohort as well as in acral melanoma (AM) and cutaneous melanoma (CM) (*P* < 0.01) (**Figure 1B**). Although no significant difference in CD68^+^ TAMs infiltration was observed between tumor and adjacent tissues in CM, it was significantly elevated in tumor tissues in the overall and AM. Further analysis of the correlation between CD68^+^ TAMs, CD163^+^ TAMs expression and clinicopathological characteristics revealed that both CD68^+^ TAMs and CD163^+^ TAMs exhibited higher infiltration levels in patients with Breslow thickness >4 mm, Clark level >III, lymph node metastasis, and AJCC stage III/IV disease (*P* < 0.01). However, no significant differences were observed among different groups stratified by age, gender, tumor surface ulceration, or surgical margin status (*P* > 0.05) (**Supplementary Tables 3, 4**).

The median follow-up time for all patients in this study was 31 months. As of December 2024, 12 of 65 patients had died due to tumor recurrence, metastasis, or related complications, while 53 were censored, mainly due to loss to follow-up, survival at the end of follow-up, or death from other causes. Kaplan-Meier survival curves were plotted with patients stratified by CD68^+^ TAMs and CD163^+^ TAMs infiltration levels, with overall survival (OS) as the endpoint. The results showed that patients in the high CD68^+^ TAMs infiltration group and high CD163^+^ TAMs infiltration group had inferior OS compared with their respective low infiltration groups (*P* < 0.05), suggesting that melanoma patients with high CD68^+^ TAMs and CD163^+^ TAMs infiltration have poor prognosis (**Figure 1C**).

Cox regression models were used to analyze factors influencing survival time. Univariate Cox regression analysis showed that age, tumor thickness, Clark level, lymph node metastasis status, CD68^+^ TAMs infiltration, CD163^+^ TAMs infiltration, and AJCC stage were associated with overall survival. Multivariate Cox regression analysis further revealed that CD163^+^ TAMs infiltration was an independent prognostic factor for survival time. The mortality risk in the low CD163^+^ TAMs infiltration group was only 0.143-fold that of the high infiltration group, indicating a significantly higher mortality risk in patients with high CD163^+^ TAMs infiltration (HR = 0.143, *P* = 0.018; **Supplementary Table 5**).

In conclusion, M2-type TAMs exhibited higher infiltration levels in melanoma tissues compared to adjacent normal tissues, and patients with high CD163^+^ TAMs infiltration demonstrated worse prognosis and higher mortality risk, suggesting that CD163^+^ TAMs may be associated with unfavorable clinical outcomes in melanoma patients.

### Melanoma cell-conditioned medium induces the polarization of macrophages toward the M2 phenotype

THP-1 cells were induced to differentiate into M0 macrophages using PMA. Microscopic observation revealed that undifferentiated THP-1 cells grew in suspension and exhibited a spherical morphology with uniform size. Following PMA induction, the cells differentiated into adherent M0 macrophages, characterized by enlarged cell bodies, abundant cytoplasm, irregular morphology, and a small number of fibroblast-like pseudopodia extending from the cell periphery(**Supplementary Figure 1A**). RT-qPCR analysis showed that CD11b mRNA expression was significantly upregulated in M0 macrophages compared with THP-1 cells(**Supplementary Figure 1B**).The polarization status of A375-TAMs and SK-TAMs induced by melanoma cell-conditioned medium was examined using RT-qPCR, Western blotting, and FCM. The results showed that compared with the M0 control group, the mRNA expression of M2-type macrophage marker genes (CD163, CD206, IL-10, Arg-1) was significantly upregulated in A375-TAMs and SK-TAMs cells, accompanied by a marked increase in the number of cells positive for CD163 and CD206 surface antibodies (*P* < 0.01) (**Figures 1D, E**). Conversely, the mRNA and protein expression of the M1-type macrophage marker gene (iNOS) was downregulated (*P* < 0.01) (**Supplementary Figures 2A, B**). These findings suggest that TAMs induced by melanoma cell-conditioned medium are polarized toward the M2 phenotype.

### HIF-1α induces M2 polarization of TAMs and enhances malignant biological behaviors of melanoma cells *in vitro*

**Figure 2:**
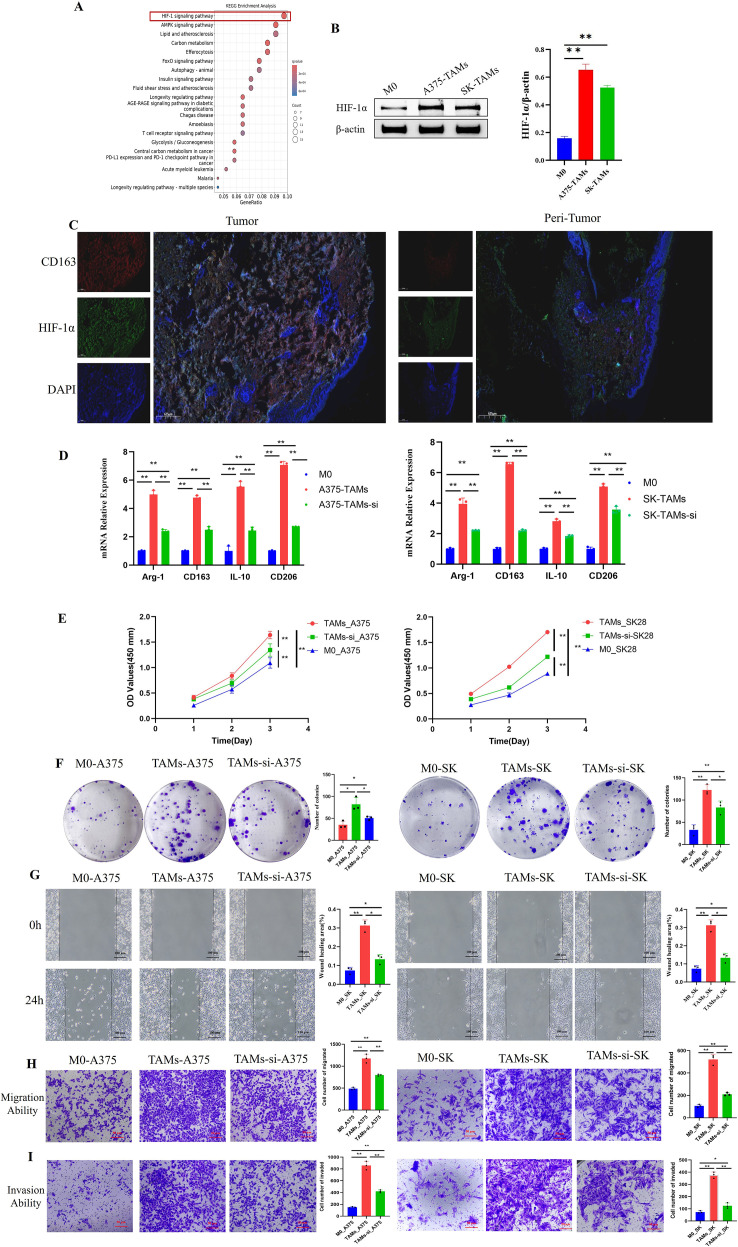
In an *in vitro* environment, HIF-1α enhances the viability, migration, invasion, and colony-forming abilities of melanoma cells by promoting the polarization of TAMs toward the M2 phenotype. **(A)** KEGG enrichment analysis of overlapping genes indicated that these genes were enriched in the HIF-1 pathway; **(B)** HIF-1α expression in TAMs was significantly higher than that in M0 macrophages(n=3 per group, error bars represent SD, and statistical analysis was performed using one-way ANOVA); **(C)** The fluorescent expression levels of HIF-1α and CD163 proteins in melanoma tissues were higher than those in adjacent tissues (scale bar = 625 μm, n=10 per group); **(D)** HIF-1α gene interference inhibited the polarization of TAMs (induced by melanoma cells) toward the M2 phenotype(n=3 per group, error bars represent SD, and statistical analysis was performed using one-way ANOVA); **(E–I)** HIF-1α gene interference downregulated the promotional effect of TAMs on melanoma cell viability **(E)**, colony-forming ability **(F)**, migration **(G, H)**, and invasion **(I)** (n=3 per group, error bars represent SD, and statistical analysis was performed using one-way ANOVA). ^*^P<0.05, ^**^P<0.01.

Studies have shown that metabolic reprogramming plays a crucial role in regulating TAMs polarization, particularly glucose metabolism. In this study, we screened for the key genes governing TAMs polarization in melanoma by leveraging melanoma scRNA-seq data and public glucose metabolism gene sets. First, clustering analysis was performed on the melanoma scRNA-seq datasets to identify the differentially expressed genes (DEGs) between M1- and M2-type TAMs (**Supplementary Figure 3A**), followed by the acquisition of the intersection between these DEGs and glucose metabolism-related signature gene sets (**Supplementary Figure 3B**). Enrichment analysis was subsequently conducted. KEGG analysis revealed the intersecting genes were significantly enriched in the HIF-1 signaling pathway (**Figure 2A**). Volcano plot analysis of the DEGs between M1- and M2-type TAMs showed that hypoxia-inducible HIF-1α and glycolysis-related regulatory genes, including pyruvate kinase M (PKM), hexokinase 2 (HK2) and 6-phosphofructo-2-kinase/fructose-2,6-bisphosphatase 3 (PFKFB3), were markedly upregulated in M2-type TAMs compared with M1-type TAMs (**Supplementary Figure 3C**; **Supplementary Table 7**). Collectively, these findings suggest that HIF-1α may play a crucial role in the polarization of TAMs in melanoma. Given that *de novo* serine synthesis pathway (SSP), a vital branch of glycolysis, exerts an important function in tumor progression, we further validated the key genes of HIF-1α, glycolysis and SSP in subsequent experiments.

Western blotting was performed to detect HIF-1α protein expression in M0 macrophages and TAMs induced by melanoma cell-conditioned medium. The results showed that HIF-1α protein expression in TAMs was significantly higher than that in M0 macrophages (**Figure 2B**). The relationship between HIF-1α and TAMs polarization was further explored by tissue IF and RT-qPCR experiments. Dual immunofluorescence staining of HIF-1α and CD163 in melanoma tissues and adjacent normal tissues showed that the expression levels of CD163 and HIF-1α proteins in tumor tissues were significantly higher than those in adjacent tissues, and the expression of CD163 and HIF-1α was highly co-localized at the tissue level (**Figure 2C**). Furthermore, lentiviral vectors were employed to knock down HIF-1α in TAMs, thereby constructing HIF-1α-silenced TAMs (TAMs-si). RT-qPCR was used to detect the mRNA expression of M2-type macrophage marker genes in M0, TAMs and TAMs-si. The results showed that after HIF-1α gene interference, the mRNA expression levels of CD163, CD206, Arg-1, and IL-10 in TAMs-si were significantly decreased (**Figure 2D**). These findings suggest that HIF-1α gene interference inhibits the polarization of TAMs toward the M2 type in melanoma.

To explore whether HIF-1α modulates the role of TAMs in regulating malignant biological behaviors of melanoma cells. A375 and SK-MEL-28 cells were treated with the cell-conditioned medium of M0 macrophages, TAMs, and TAMs-si. CCK-8 assay, wound healing assay, migration assay, invasion assay, and colony formation assay were then performed. The results showed that compared with the M0 macrophage group, tumor cells in the TAMs group exhibited increased cell viability, enhanced migration, invasion, and colony-forming ability. However, after HIF-1α gene interference, the cell viability (**Figure 2E**), colony formation ability (**Figure 2F**), migration ability (**Figures 2G, H**), and invasion ability (**Figure 2I**) in the TAMs-si group were significantly lower than those in the TAMs group. These findings indicate that *in vitro* environment, interfering with HIF-1α can attenuate the role of TAMs in promoting the malignant biological behaviors of melanoma cells.

### HIF-1α promotes melanoma progression in nude mice by inducing TAMs polarization toward the M2 phenotype

**Figure 3:**
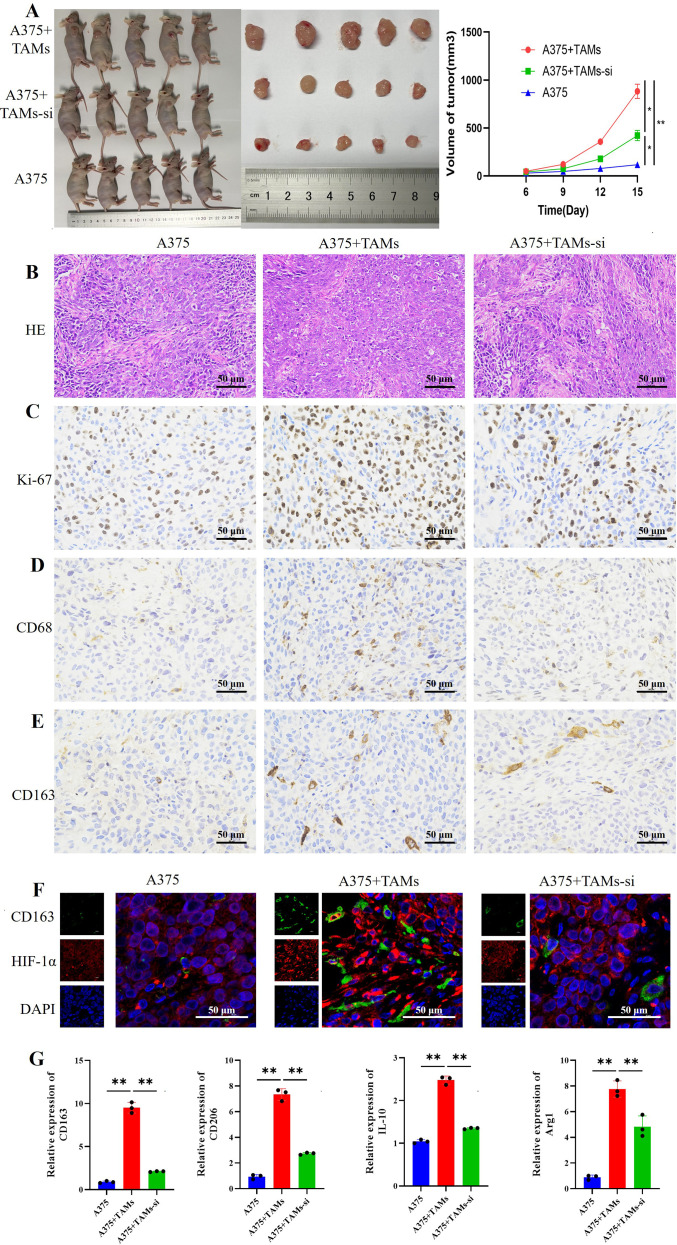
Interference with the HIF-1α gene inhibits the polarization of TAMs toward the M2 subtype and the pro-tumor effect of TAMs in subcutaneous xenografts of nude mice. **(A)** *In vivo* and ex vivo images, volume change trends (n=5 per group, and statistical analysis was performed using one-way ANOVA,; **(B)** HE staining images (scale bar = 100μm); **(C–G)** Interference with the HIF-1α gene reduces Ki-67 expression **(C)**, the infiltration levels of CD68^+^ TAMs **(D)** and CD163^+^ TAMs **(E)**, the mRNA expression of M2-type macrophage marker genes **(G)**, n=3 per group, error bars represent SD, and statistical analysis was performed using one-way ANOVA),; **(F)** HIF-1α expression in CD163^+^ M2-type TAMs in subcutaneous xenografts of nude mice (scale bar = 50μm, n=3 per group). ^*^P<0.05, ^**^P<0.01.

To verify the effect of HIF-1α on the pro-tumor function of TAMs *in vivo*, a nude mouse subcutaneous xenograft model was established with three groups. The results showed that the subcutaneous tumor volume in the A375+TAMs co-injection group was the largest with the fastest growth rate, followed by the A375+TAMs-si group. The A375-only injection group had the smallest tumor volume and the slowest growth rate (**Figure 3A**). HE staining and Ki-67 staining of subcutaneous xenografts showed that in the A375+TAMs group, the tumor tissue presented with indistinct boundaries, abundant TAMs were observed, and Ki-67 expression was the highest; compared with the A375+TAMs group, the A375+TAMs-si group exhibited reduced TAMs count in tumor tissue, distinct tumor boundaries, and decreased Ki-67 expression; in the A375-only injection group, tumor cells were closely arranged, the tumor tissue had the most distinct boundaries, and Ki-67 expression was the lowest (**Figures 3B, C**). These results indicate that TAMs can promote tumor tissue invasion and tumor cell proliferation *in vivo*; however, this pro-tumorigenic capacity is significantly attenuated following HIF-1α knockdown.

IHC and RT-qPCR analyses of subcutaneous xenografts in nude mice demonstrated that the infiltration levels of CD68^+^ TAMs and CD163^+^ TAMs, along with the mRNA levels of M2 macrophage marker genes (CD163, CD206, IL-10, Arg-1), were highest in the A375+TAMs group, followed by the A375+TAMs-si group and the A375-only injection group; the A375+TAMs group had significantly higher levels than the A375+TAMs-si group and the A375 alone group (**Figures 3D, E, G**). Additionally, immunofluorescence co-staining for HIF-1α and CD163 was performed in subcutaneous xenograft tissues derived from the three nude mouse groups. The results showed that CD163 and HIF-1α were weakly expressed in tumor tissues from the A375 group; CD163 exhibited the highest expression in the A375+TAMs group, and cells with high HIF-1α expression also showed elevated CD163 expression. In contrast, the expression levels of both CD163 and HIF-1α in the A375+TAMs-si group were significantly decreased compared with those in the A375+TAMs group (**Figure 3F**). These results indicate that *in vivo*, HIF-1α gene interference suppresses the polarization of TAMs to the M2 subtype and impedes tumor progression.

### The expression of key glucose-serine metabolic enzymes is upregulated in melanoma TAMs, and is downregulated after HIF-1α interference

Since glucose metabolic reprogramming plays an important role in the polarization of TAMs, our study detected the expression of GLUT1 and PKM2 in melanoma TAMs. These two enzymes play critical roles in glucose metabolism, they can maintain cellular energy and biosynthetic homeostasis and regulate the direction of glucose metabolism (27, 28). In addition, SSP is an important branch of glycolysis, and existing studies suggest that serine may also be involved in macrophages polarization (15, 16). Our study also detected PHGDH, PSAT1, and PSPH, the key rate-limiting enzymes in *de novo serine synthesis*, which can convert the glycolytic intermediate 3-phosphoglycerate(3-PG) into serine (13).

**Figure 4:**
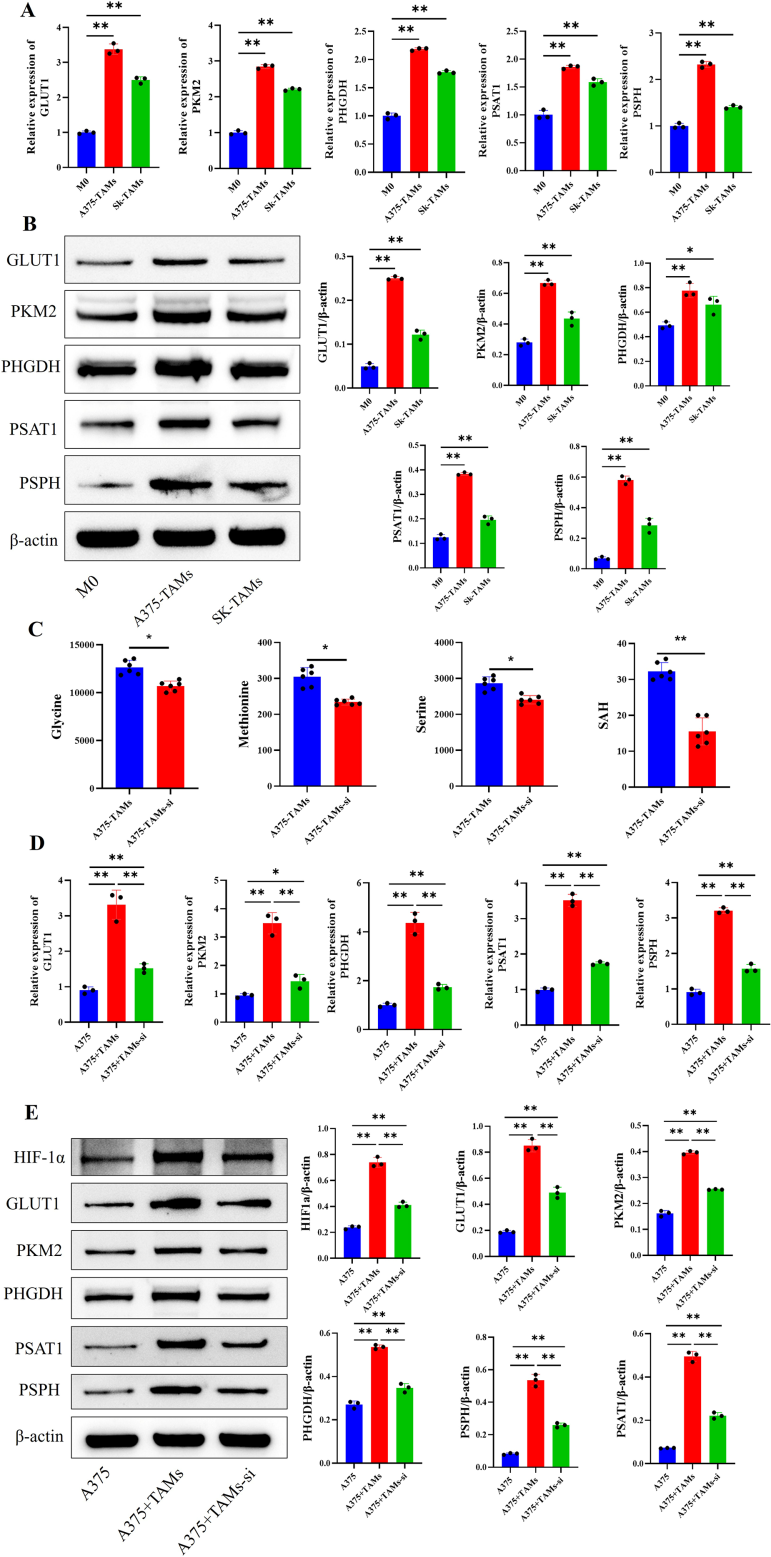
The expression of key glucose-serine metabolic enzymes is upregulated in melanoma TAMs and is downregulated after HIF-1α gene interference. **(A, B)** The mRNA **(A)** and protein **(B)** expressions of key glucose-serine metabolic enzymes are upregulated in TAMs induced by melanoma cell supernatants (n=3 per group, error bars represent SD, and statistical analysis was performed using one-way ANOVA); **(C)** The contents of Glycine, Methionine, Serine, and SAH in TAMs are downregulated after HIF-1α interference (n=6 per group, error bars represent SD, and statistical analysis was performed using one-way ANOVA,; **(D, E)** HIF-1α gene interference downregulates the mRNA **(D)** and protein **(E)** expressions of key glucose-serine metabolic enzymes in nude mouse subcutaneous xenografts (n=3 per group, error bars represent SD, and statistical analysis was performed using one-way ANOVA). ^*^P<0.05, ^**^P<0.01.

This study detected the expression levels of key glucose-serine metabolic enzymes *in vitro*-induced TAMs. RT-qPCR and Western blot results showed that compared with M0 macrophages, the mRNA and protein expressions of key glucose-serine metabolic enzymes (GLUT1, PKM2, PHGDH, PSAT1, and PSPH) in A375-TAMs and SK-TAMs were significantly increased. These results suggest that glucose-serine metabolism is upregulated in TAMs induced by melanoma cell-conditioned medium (**Figures 4A, B**).

Further, amino acid metabolomics was used to detect differential amino acids in A375-TAMs and A375-TAMs-si cells, the results showed that the serine content in the A375-TAMs-si group was significantly lower than that in the A375-TAMs group (**Figure 4C**); RT-qPCR and Western blotting were performed to detect the expression levels of key glucose-serine metabolic enzymes in subcutaneous xenograft tumors of nude mice in the three groups. The results showed that the mRNA and protein expressions of GLUT1, PKM2, PHGDH, PSAT1, and PSPH was the lowest in the A375-only injection group; compared with the A375+TAMs group, the mRNA and protein expressions of key glucose-serine metabolic enzymes in the A375+TAMs-si group were decreased (**Figures 4D, E**). These results suggest that HIF-1α interference downregulates glucose-serine metabolism levels in TAMs both *in vitro* and *in vivo*.

### HIF-1α knockdown inhibits the polarization of TAMs toward the M2-type in melanoma by downregulating glucose-serine metabolism

**Figure 5:**
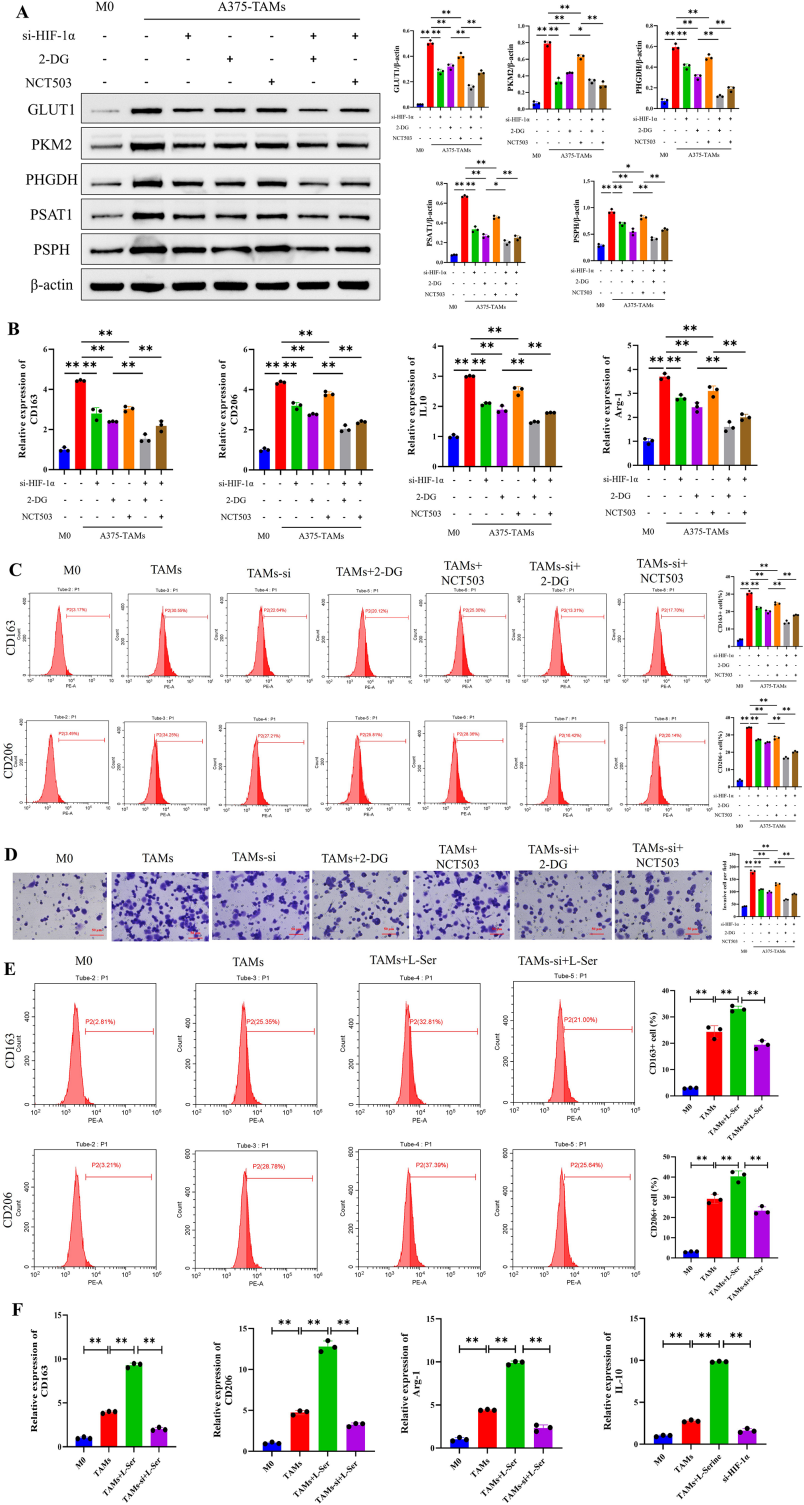
In melanoma, HIF-1α promoted the polarization of TAMs toward the M2 phenotype by regulating glucose-serine metabolism, and exogenous serine also promoted the polarization of TAMs toward the M2 phenotype. **(A)** Western blot showed that HIF-1α interference downregulated the protein expression of key glucose-serine metabolic enzymes in TAMs induced by melanoma cell supernatant. **(B)** RT-qPCR indicated that HIF-1α gene interference downregulated the mRNA expression of M2-type macrophage marker genes in TAMs. **(C)** FCM demonstrated that HIF-1α gene interference reduced the proportion of M2-type macrophages in TAMs. **(D)** Transwell invasion assay revealed that HIF-1α gene interference decreased the invasive capacity of TAMs. **(E)** FCM showed that exogenous serine increased the proportion of M2-type macrophages in TAMs. **(F)** RT-qPCR demonstrated that exogenous serine upregulated the mRNA expression of M2-type marker genes in TAMs. (n=3 per group, error bars represent SD, and statistical analysis was performed using one-way ANOVA, ^**^P<0.01, ^*^P<0.05).

To clarify whether HIF-1α induces macrophage polarization by regulating glucose-serine metabolism, TAMs were treated with 2-DG (a glucose metabolism inhibitor) or NCT503 (a serine metabolism inhibitor) in this study. Subsequently, the protein expression levels of key glucose-serine metabolic enzymes, including GLUT1, PKM2, PHGDH, PSAT1, and PSPH, were detected in A375-TAMs from each group. The results showed that expression of key glucose-serine metabolic proteins was the lowest in M0 macrophages and the highest in the control A375-TAMs group. In comparison, the protein expression levels in A375-TAMs groups with HIF-1α interference, 2-DG treatment, or NCT503 treatment alone were all lower than those in the untreated A375-TAMs group. In addition, CCK-8 results indicated that 1000 μM 2-DG and 10 μM NCT503 used in this study had no effect on TAM viability ((**Supplementary Figure 4**). Furthermore, combined treatment of HIF-1α knockdown with 2-DG or NCT503 led to lower expression levels of these key metabolic enzymes compared with the groups treated with 2-DG or NCT503 alone (**Figure 5A**). Collectively, these results indicate that HIF-1α knockdown, 2-DG treatment, and NCT503 treatment can all downregulate the expression of key glucose-serine metabolic enzymes in A375-TAMs.

The expression of M2 macrophage polarization markers in A375-TAMs from each group was determined by RT-qPCR and FCM. The results showed that, compared with M0 macrophages, the untreated A375-TAMs group exhibited a significant elevation in both the mRNA expression levels of M2-specific marker genes (CD163, CD206, IL-10, and Arg-1) and the proportion of CD163^+^ and CD206^+^ M2 macrophages. In contrast, HIF-1α knockdown, 2-DG treatment, or NCT503 treatment alone resulted in a marked reduction in M2 macrophage polarization markers in A375-TAMs relative to the untreated group. Furthermore, combined treatment with HIF-1α knockdown in combination with 2-DG or NCT503 led to a further decrease in these M2 polarization markers (**Figures 5B, C**). Additionally, HIF-1α knockdown, 2-DG treatment, or NCT503 treatment alone attenuated the invasive capacity of A375-TAMs (**Figure 5D**). Collectively, these results demonstrate that HIF-1α knockdown can downregulate glucose-serine metabolism in TAMs, thereby inhibiting M2 polarization and reducing the invasive capacity of TAMs.

### Exogenous serine promotes the polarization of TAMs toward the M2 phenotype

To further investigate whether exogenous serine promotes the polarization of TAMs toward the M2 phenotype, exogenous serine was added during the polarization of TAMs induced by melanoma cell-conditioned medium. RT-qPCR and FCM results showed the following: compared with the TAMs group, the TAMs+L-ser group had increased mRNA expression of M2 macrophage marker genes (CD163, CD206, IL-10, Arg-1) and a higher proportion of CD163^+^ and CD206^+^ M2 macrophages; however, when HIF-1α was knocked down in the presence of exogenous serine (TAMs-si+L-ser), the expression of M2 macrophage markers was downregulated (**Figures 5F, G**). These results suggest that exogenous serine can induce the polarization of melanoma-associated TAMs toward the M2 phenotype, and HIF-1α acts as an upstream factor to regulate serine metabolism and inducing TAMs polarization.

### PKM2/HIF-1α promotes M2 polarization of TAMs by regulating glucose-serine metabolism

**Figure 6:**
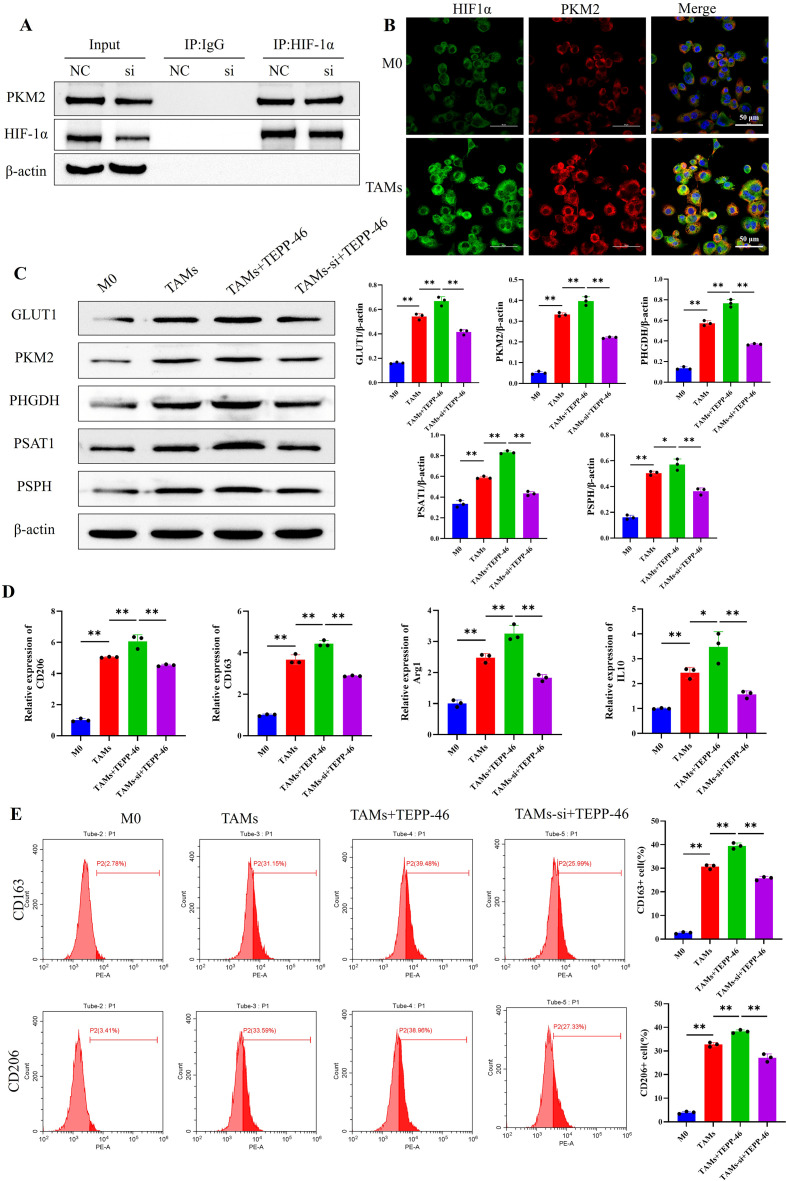
The PKM2/HIF-1α axis upregulates glucose-serine metabolism to induce M2 polarization of TAMs. **(A)** The Co-IP assay indicates that there is a protein interaction between HIF-1α and PKM2 in TAMs; **(B)** Cellular IF shows that HIF-1α and PKM2 are colocalized at the single-cell level in TAMs (scale bar = 50μm); **(C)** Western blot reveals that the PKM2/HIF-1α axis upregulates the expression of key glucose-serine metabolic enzyme proteins in TAMs; **(D, E)** The PKM2/HIF-1α axis upregulates the mRNA expression of M2 macrophage marker genes **(D)** and the proportion of M2 macrophages **(E)** in TAMs. (n=3 per group, error bars represent SD, and statistical analysis was performed using one-way ANOVA, ^*^P<0.05, ^**^P<0.01).

Studies suggest that PKM2 and HIF-1α have a mutual regulatory relationship. To verify whether this regulation exists in melanoma-induced TAMs, this study used Co-IP to detect the protein interaction between HIF-1α and PKM2 in TAMs. The results showed that PKM2 could be detected in HIF-1α-enriched proteins (**Figure 6A**). Immunofluorescence staining showed that the expression of HIF-1α and PKM2 in TAMs were significantly higher than those in M0 macrophages, and HIF-1α and PKM2 were co-expressed at the single-cell level (**Figure 6B**). These results indicate that there is a reciprocal regulatory relationship between PKM2 and HIF-1α in melanoma-induced TAMs.

Further, TEPP-46 was used to activate the PKM2 gene, and a rescue experiment was conducted by activating PKM2 while interfering with the HIF-1α gene. Results from Western blot, RT-qPCR, and FCM showed that after PKM2 gene activation, the expression of key glucose-serine metabolic enzyme proteins, the mRNA expression of M2 macrophage marker genes, and M2 macrophage proportion in the TAMs+TEPP-46 group were all upregulated compared with the TAMs group. However, when the HIF-1α gene was simultaneously interfered with, the expression of the above substances in the TAMs-si+TEPP-46 group was downregulated (**Figures 6C–E**). Combined with the above results, it is suggested that the PKM2 gene is upstream of HIF-1α in TAMs; The PKM2/HIF-1α axis induced the polarization of TAMs toward the M2 phenotype by upregulating the expression of key enzymes involved in glucose-serine metabolism.

**Figure 7:**
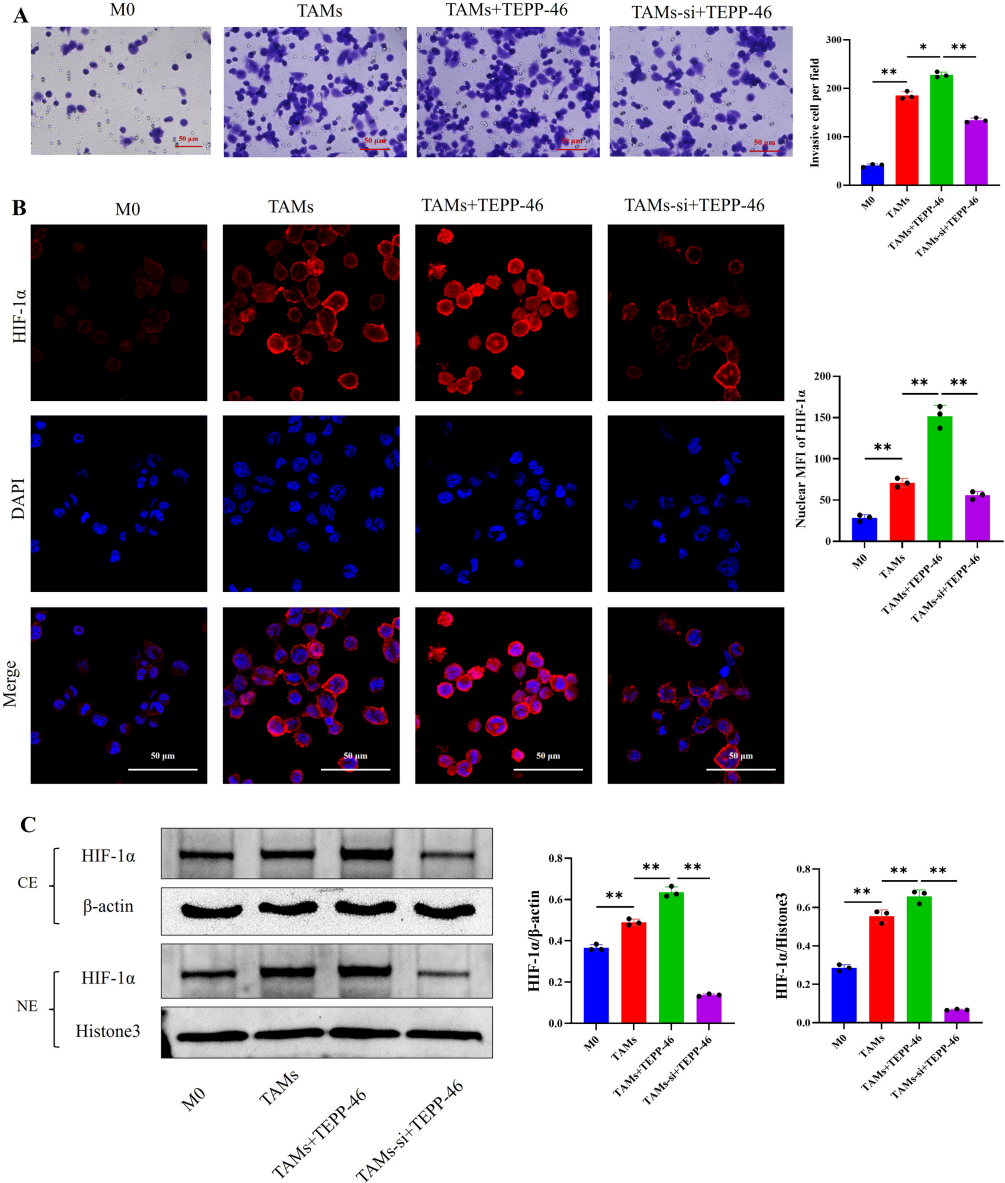
The PKM2/HIF-1α axis upregulates the invasive ability of TAMs and the expression of HIF-1α in the cell nucleus; **(A)** The Transwell invasion assay indicates that the PKM2/HIF-1α axis upregulates the invasive ability of TAMs; **(B)** Cellular IF shows that the PKM2/HIF-1α axis upregulates the expression of HIF-1α in the nucleus of TAMs (scale bar = 50μm); **(C)** Western blot reveals that the PKM2/HIF-1α axis upregulates the protein expression of HIF-1α in the nucleus of TAMs. (n=3 per group, error bars represent SD, and statistical analysis was performed using one-way ANOVA, ^*^P<0.05, ^**^P<0.01).

The Transwell invasion assay was used to detect the invasive ability of TAMs in each group. The results showed that the number of macrophages that migrated through the membrane in the TAMs+TEPP-46 group was higher than that in the TAMs group; however, when the downstream HIF-1α gene was interfered with, the number of migrated TAMs decreased significantly (**Figure 7A**). This suggests that the PKM2/HIF-1α axis can regulate the invasive ability of TAMs.

Cellular IF staining was performed to determine the subcellular localization of HIF-1α in TAMs from each group, whereas Western blot assay was used to detect the protein expression levels of HIF-1α in the nuclear and cytoplasmic fractions of TAMs across all groups. The results showed that after activating the PKM2 gene, the expression of HIF-1α in the cell nuclei of the TAMs+TEPP-46 group was upregulated; however, after interfering with the downstream HIF-1α gene, the expression of HIF-1α in the cell nuclei was downregulated(**Figures 7B, C**). Together with our previous findings, these results suggest that the PKM2/HIF-1α axis can induce the polarization of TAMs toward the M2 phenotype by promoting the nuclear translocation of HIF-1α.

### A self-reinforcing circuit exists among PKM2/HIF-1α/serine in TAMs

**Figure 8:**
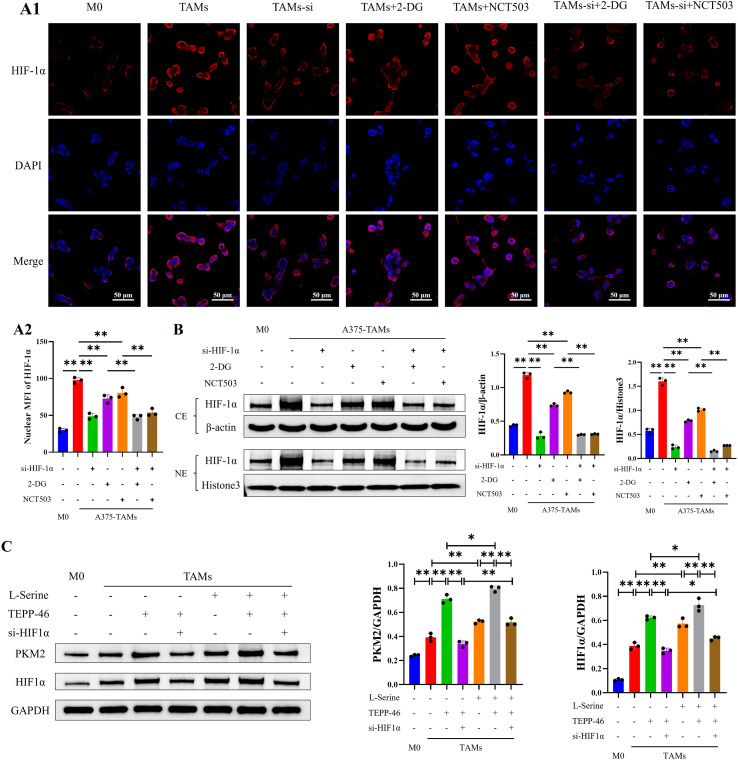
Positive feedback relationship among PKM2, HIF-1α, and serine in TAMs. **(A)** Cellular IF results indicated that inhibiting glucose and serine metabolism could reduce the expression of HIF-1α in TAMs (scale bar = 50μm); **(B)** Western blot analysis indicated that inhibiting glucose and serine metabolism reduced HIF-1α expression in the nucleus of TAMs; **(C)** Exogenous serine upregulated the protein expression of PKM2 and HIF-1α in TAMs;(n=3 per group, error bars represent SD, and statistical analysis was performed using one-way ANOVA. ^*^P<0.05, ^**^P<0.01).

In this study, IF and Western blot were used to detect the subcellular localization of HIF-1α and nuclear HIF-1α expression in TAMs treated with HIF-1α knockdown, 2-DG, or NCT503. The results showed that compared with the TAMs group, the expression of HIF-1α in the nucleus was significantly decreased in the groups with HIF-1α interference or treated with 2-DG or NCT503. This indicates that inhibiting glucose or serine metabolism can also feedback inhibit the expression of HIF-1α in the nucleus, suggesting a potential reciprocal regulation between glucose-serine metabolism and HIF-1α (**Figures 8A, B**).

**Figure 9:**
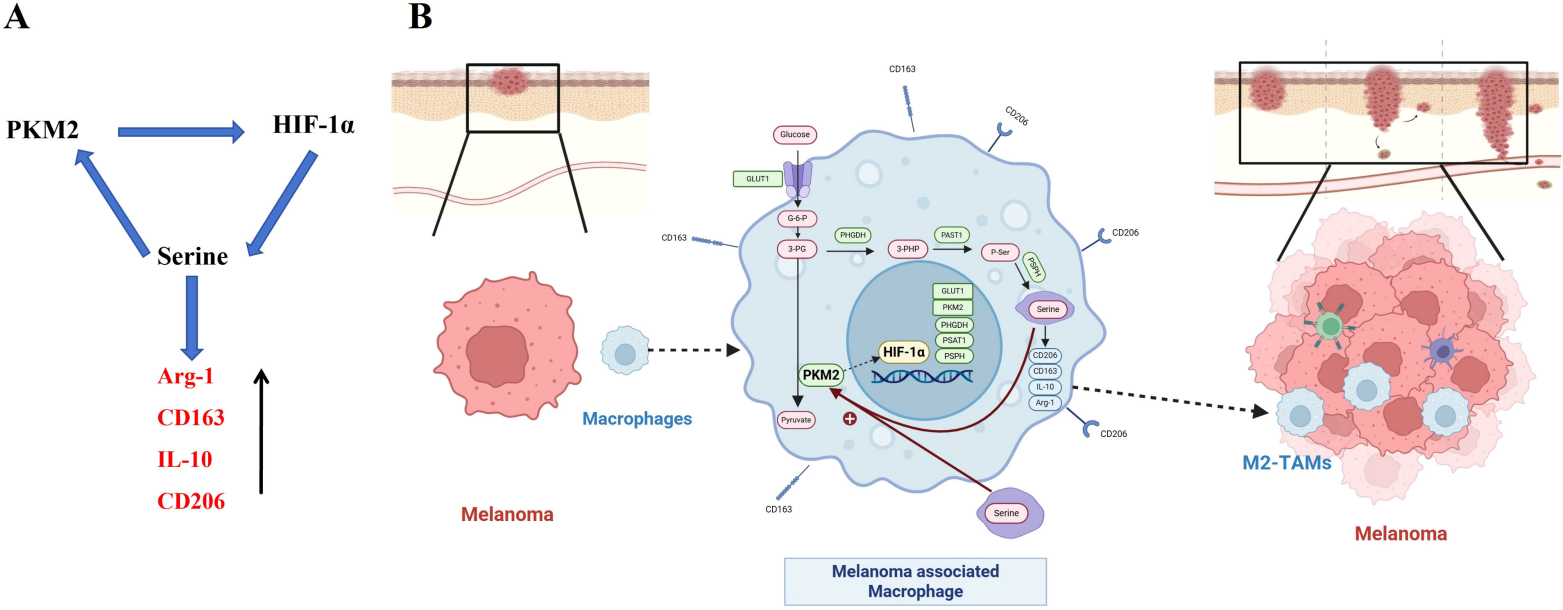
Schematic diagram of the PKM2/HIF-1α axis promoting melanoma progression by polarizing TAMs via upregulating glucose-serine metabolism **(A)** Diagram of the positive feedback regulatory relationship of the PKM2/HIF-1α/serine axis in TAMs. **(B)** Schematic diagram of the proposed mechanism in this study.

Previous studies have indicated that serine can activate PKM2 by binding to the allosteric site of PKM2 and enhancing its enzymatic activity, yet this phenomenon has been rarely reported in TAMs. Western blot was employed to detect the protein expressions of PKM2 and HIF-1α in TAMs of each group after the addition of exogenous serine. The results showed that PKM2 activation upregulated the protein expressions of PKM2 and HIF-1α in TAMs; after adding exogenous serine, the protein expressions of PKM2 and HIF-1α were significantly higher than those in the TAMs group, indicating that serine can upregulate the protein expression levels of PKM2 and HIF-1α in TAMs (**Figure 8C**). These results demonstrate that in melanoma-associated TAMs, PKM2 can upregulate the HIF-1α, and HIF-1α subsequently promotes SSP in TAMs. In turn, serine can further activate the PKM2, thereby forming a positive feedback regulatory loop among PKM2, HIF-1α, and serine. Ultimately, the elevated serine levels induce the polarization of melanoma-associated TAMs toward the M2 phenotype, thereby exerting pro-tumorigenic effects (**Figure 9A**).

## Discussion

Melanoma is a malignant tumor originating from melanocytes, characterized by high malignancy, rapid progression, high metastatic potential, a continuous rise in incidence, and suboptimal therapeutic efficacy (29–34). Therefore, in-depth elucidation of the regulatory mechanisms of melanoma and inhibition of its progression are core issues urgently needing resolution in the current field of melanoma treatment. As key regulators in the TME, TAMs promote tumor progression through multiple aspects, including enhancing tumor invasion and metastasis, inducing angiogenesis, and strengthening cancer stemness. In this study, clinical sample analysis revealed that M2-type TAMs exhibited significantly higher infiltration in melanoma tumor tissues compared to adjacent normal tissues, a finding consistent with the expression pattern of TAMs observed in various solid tumors in previous studies (35, 36). Further analysis demonstrated that patients with high CD163^+^ TAMs infiltration exhibited worse prognosis and higher mortality risk. Although the relatively limited sample size and low event rate represent limitations of this study, our findings are consistent with the conclusions of several large-scale studies (37–39), all of which indicate that M2-type TAMs are associated with poor prognosis in melanoma patients, thereby providing robust clinical and experimental evidence supporting the reliability of our conclusions.

TAMs are the largest consumers of glucose at the single-cell level in the TME (31); thus, glucose metabolism exerts a critical influence on TAMs polarization. Studies have revealed the upregulation of glycolysis-related proteins in TAMs from thyroid cancer, and pancreatic ductal adenocarcinomas (PDAC) (40). Additionally, the glycolytic inhibitor 2-DG can reduce the expression of CD163 on the TAMs surface and inhibit tumor progression (13), which collectively suggests that glycolysis is involved in the polarization process of TAMs in some tumors. In this study, we found that the expression levels of GLUT1 and PKM2—key enzymes in glucose metabolism—were upregulated in melanoma-associated TAMs; moreover, treatment with 2-DG inhibited the polarization of TAMs toward the M2 phenotype, indicating that glycolysis also participates in the M2 polarization of melanoma-associated TAMs.

SSP, a key branch of glycolysis, is closely associated with glycolysis. As a critical source of one-carbon units, serine is involved in the regulation of various tumor cells and T lymphocytes (41–44), and has been primarily studied in tumor cells (45, 46). However, research on the involvement of serine metabolism in TAMs polarization remains limited, and the existing findings are inconsistent. Cai et al. treated PHGDH-deficient macrophages with conditioned media from human lung cancer and colon cancer cells, and observed decreased expression of the M2 macrophage markers ARG1 and TGF-β (16). Poczobutt et al. found that PHGDH expression was significantly upregulated in TAMs isolated from mouse orthotopic lung tumors (47). Additionally, studies have suggested that exogenous serine promotes M2 type TAMs polarization and tumor progression (48). In contrast, other research has shown that serine metabolism is downregulated in breast cancer TAMs, and inhibition of PHGDH promotes the conversion of TAMs to an M2-like phenotype (49). In the present study, our results demonstrated that the expression of key metabolic enzymes in the SSP—PHGDH, PSAT1, and PSPH—was upregulated in melanoma-associated TAMs, both *in vitro* and *in vivo*. Interference with the SSP inhibited the polarization of melanoma-associated TAMs toward the M2 phenotype, while supplementation with exogenous serine promoted this M2 polarization. These findings indicate that the SSP facilitates the M2 polarization of melanoma-associated TAMs. The differences in serine metabolism alterations among TAMs in different tumors may be attributed to tumor heterogeneity.

HIF-1α is a key transcription factor in hypoxic environments that regulates metabolic homeostasis in various cell types, including tumor cells and macrophages, thereby inducing metabolic reprogramming. Our results demonstrated that knockdown of HIF-1α significantly downregulated the mRNA and protein expression levels of key glycolytic and SSP metabolic enzymes (GLUT1, PKM2, PHGDH, PSAT1, and PSPH) in melanoma TAMs, indicating that HIF-1α regulates glucose-serine metabolism in TAMs. Combined with the regulatory effects of serine metabolism on TAMs polarization and rescue experiments, we speculated that HIF-1α transcriptionally regulates key enzymes involved in glucose-serine metabolism and upregulates their expression, thereby enhancing glycolysis and SSP. The elevated serine levels further drive the polarization of TAMs toward the pro-tumorigenic M2 phenotype. Current studies on HIF-1α-mediated metabolic regulation in TAMs have primarily focused on glucose metabolism (9–13), our findings reveal that, beyond glucose, HIF-1α can also promote M2 polarization of TAMs in melanoma by regulating SSP. This discovery represents a valuable addition to the understanding of HIF-1α-mediated serine metabolic regulation and associated polarization functions in tumor-associated macrophages.

PKM2, a key glycolytic enzyme, exhibits a mutual regulatory relationship with HIF-1α. Previous studies have demonstrated that HIF-1α can directly activate PKM2 gene transcription (50, 51). PKM2 can also promote HIF-1α expression,PKM2 translocates to the nucleus as a dimer through acetylation and phosphorylation modifications, recruiting histone acetyltransferase p300 to enhance HIF-1α transactivation capacity (52, 53). In this study, Co-IP, IF, and functional rescue experiments confirmed that PKM2 is an upstream regulator of HIF-1α. The regulatory role of the PKM2/HIF-1α axis in inducing TAMs polarization through glucose-serine metabolism suggests that targeting this axis to downregulate glucose-serine metabolism and inhibit TAMs polarization may represent a promising therapeutic strategy for melanoma treatment.

Additionally, our study found that treatment with 2-DG and NCT503 to inhibit serine metabolism resulted in downregulated HIF-1α expression in the melanoma-induced TAMs, suggesting that inhibition of glucose-serine metabolism can also interfere with HIF-1α expression. Furthermore, supplementation with exogenous serine upregulated PKM2 and HIF-1α protein expression in TAMs, indicating that serine can enhance PKM2/HIF-1α expression in melanoma TAMs. Previous studies has identified serine as a natural ligand and allosteric activator of PKM2: it binds to the allosteric site of PKM2, promoting the conversion of PKM2 from the low-activity dimeric form to the high-activity tetrameric form (25) and thus enhancing the expression of the PKM2 gene. Notably, the activating effect of serine on PKM2 has only been reported in tumor cells and vascular endothelial cells to date (54, 55). In this study, we further identified a positive feedback regulatory loop among PKM2, HIF-1α and serine in melanoma TAMs, which extends and enriches the current understanding of this regulatory network in the field.

Owing to time and tissue sample constraints, the present study has certain limitations. First, he patient tissue specimens analyzed in this research were exclusively obtained from Chinese melanoma patients, and the expression levels of PKM2 and key enzymes involved in the SSP were not examined in melanoma tissues with high CD163^+^ TAM infiltration. additionally, The limited sample size and low event rate in this study have imposed certain constraints on the survival analysis results; however, our conclusions are consistent with the findings of Jensen TO et al. (38). Second, regarding genetic regulatory relationships, although we identified that the PKM2/HIF-1α axis induces M2 polarization of melanoma-associated TAMs by upregulating glucose-serine metabolism, we did not verify the role of upstream PKM2 through genetic knockdown assays. Finally, given that TAMs exhibit a high degree of plasticity and heterogeneity *in vivo*, their phenotypic spectrum represents a dynamic continuum rather than two strictly differentiated polar states. Therefore, the “M2-type” TAMs defined by the expression of CD163, CD206, IL-10 and Arg-1 in this study should be interpreted as an M2-like phenotype, which merely represents a polarized tendency toward the M2 state, rather than a functionally terminally differentiated or definitive macrophage subtype. In our subsequent studies, we will incorporate more specific markers and functional detection indicators to more comprehensively elucidate the specific mechanism by which the PKM2/HIF-1α axis induces TAMs polarization in melanoma through the regulation of glucose-serine metabolism.

## Conclusion

M2-type TAMs play a role in promoting melanoma progression. As a key factor regulating glucose metabolism, HIF-1α can induce the polarization of TAMs toward the M2 phenotype. In TAMs, the PKM2 gene enhances glucose metabolism and SSP by transactivating HIF-1α, thereby promoting TAMs polarization toward the M2 phenotype. Additionally, SSP in TAMs and exogenously absorbed serine can jointly enter the PKM2/HIF-1α/serine positive feedback regulatory axis, further promoting TAMs polarization toward the M2 phenotype and thus exerting pro-tumor effects (**Figure 9B**). During the above regulatory process, the PKM2/HIF-1α axis is a key mechanism for inhibiting TAMs metabolic regulation and polarization. Targeting the PKM2/HIF-1α axis is expected to provide a new potential direction for melanoma treatment.

## Data Availability

Publicly available datasets were analyzed in this study. This data can be found here: https://www.ncbi.nlm.nih.gov/geo/.
