# Supplementary material for: The mechanism of PKM2/HIF-1α axis polarizing TAMs by upregulating glucose-serine metabolism to promote melanoma progression
Source: Front Immunol. 2026 Mar 31;17:1740155. doi: 10.3389/fimmu.2026.1740155 (PMC13076481; doi:10.3389/fimmu.2026.1740155)
Supplement: Supplementary file 1 [file Table1.docx]

Supplementary Material

| Table S1 Primer sequences used in this study | | | |
| --- | --- | --- | --- |
| **Gene** | **Primer** | **Sequence (5'-3')** | **PCR Products** |
| Homo β-actin | Forward | GCACTCTTCCAGCCTTCCTT | 200 bp |
|  | Reverse | TTCATTGTGCTGGGTGCCA |  |
| Homo PHGDH | Forward | TCCAAGGCACTACGCCTGTA | 198 bp |
|  | Reverse | CCCCATCTGACACCAGTGAA |  |
| Homo PSAT1 | Forward | TCTACGTCATGGGCTTGGTTC | 159 bp |
|  | Reverse | TCTTGCTTCTATTTTGGGGCTC |  |
| Homo GLUT1 | Forward | GGGCATGTGCTTCCAGTATG | 178 bp |
|  | Reverse | CTTGTCACTTTGGCTGGCTC |  |
| Homo PKM2 | Forward | GGCCATAATCGTCCTCACCA | 143 bp |
|  | Reverse | ACAGCACAGGGAAGATGCC |  |
| Homo PSPH | Forward | GGTTTTGATGAGACGCAGCC | 145 bp |
|  | Reverse | AAGCATCAGCAGGAGGACAG |  |
| Homo CD206 | Forward | GGGACGTGGCTGTGGATAAA | 269 bp |
|  | Reverse | ATGATGACTACTCCGGCCAC |  |
| Homo CD163 | Forward | GAGACTGTTAGGGAAGGTG | 131 bp |
|  | Reverse | TGTTTGTTGCCTGGATT |  |
| Homo IL-10 | Forward | TGGAGGACTTTAAGGGTT | 104 bp |
|  | Reverse | GATGTCTGGGTCTTGGTT |  |
| Homo ARG1 | Forward | CTTTGCTGACATCCCTAAT | 114 bp |
|  | Reverse | CTTCCGTTCTTCTTGACTT |  |
| Homo iNOS | Forward | AGGGACAAGCCTACCCCTC | 157 bp |
|  | Reverse | CTCATCTCCCGTCAGTTGGT |  |
| Homo CD11b | Forward | ATGTCCAGAAGAGCACACGG | 180 bp |
|  | Reverse | AGGGTCTCACAAGTCTGGGT |  |

| Table S2 Antibodies used in Western blotting | | | |
| --- | --- | --- | --- |
| Product Name | Brand | Catalog Number | Dilution Ratio |
| GLUT1 antibody | Proteintech | 21829-1-AP | 1:5000 |
| PHGDH antibody | Proteintech | 14719-1-AP | 1:3000 |
| PSAT1 antibody | Proteintech | 10501-1-AP | 1:3000 |
| PSPH antibody | Proteintech | 14513-1-AP | 1:2000 |
| PKM2 antibody | Proteintech | 15822-1-AP | 1:1000 |
| iNOS antibody | Proteintech | 22226-1-AP | 1:1000 |
| β-actin antibody | Proteintech | 66009-1-Ig | 1:10000 |
| anti-HIF-1α antibody | Proteintech | 20960-1-AP | 1:60000 |
| mouse secondary antibodies | Proteintech | SA00001-1 | 1:20000 |
| rabbit secondary antibodies | Proteintech | SA00001-2 | 1:20000 |

| Table S3 The relationship between the expression of CD68^+^ TAMs in cutaneous melanoma and clinical pathological features | | | | | |
| --- | --- | --- | --- | --- | --- |
| Characteristics | CD68 | | | Z | *P* |
|  | Negative | Low | High |  |  |
| Age |  |  |  | -1.663 | 0.096 |
| ＜60 | 2 | 21 | 6 |  |  |
| ≥60 | 1 | 21 | 14 |  |  |
| Gender |  |  |  | -0.331 | 0.741 |
| Male | 1 | 20 | 8 |  |  |
| Famale | 2 | 22 | 12 |  |  |
| Ulceration |  |  |  | -0.199 | 0.842 |
| Absent | 3 | 22 | 13 |  |  |
| Present | 0 | 20 | 7 |  |  |
| Surgical margins |  |  |  | -0.913 | 0.361 |
| Negative | 3 | 40 | 18 |  |  |
| Positive | 0 | 2 | 2 |  |  |
| Tumor thickness |  |  |  | -4.909 | **＜0.001**** |
| ≤4 mm | 3 | 31 | 2 |  |  |
| ＞4mm | 0 | 11 | 18 |  |  |
| Clark level |  |  |  | -4.920 | **＜0.001**** |
| Ⅰ-Ⅲ | 3 | 38 | 6 |  |  |
| Ⅳ-Ⅴ | 0 | 4 | 14 |  |  |
| Lymph node metastasis |  |  |  | -3.852 | **＜0.001**** |
| Absent | 3 | 23 | 2 |  |  |
| Present | 0 | 19 | 18 |  |  |
| AJCC stage |  |  |  |  |  |
| I / II | 2 | 23 | 1 | -3.758 | **＜0.001**** |
| III /I V | 1 | 19 | 19 |  |  |
| Note: ***P*＜0.01 | | | | | |

| Table S4 The relationship between the expression of CD163^+^ TAMs in cutaneous melanoma and clinical pathological features | | | | | |
| --- | --- | --- | --- | --- | --- |
| Characteristics | CD163 | | | Z | *P* |
|  | Negative | Low | High |  |  |
| Age |  |  |  | -1.420 | 0.156 |
| ＜60 | 2 | 24 | 3 |  |  |
| ≥60 | 1 | 27 | 8 |  |  |
| Gender |  |  |  | -.0221 | 0.825 |
| Male | 1 | 23 | 5 |  |  |
| Famale | 2 | 28 | 6 |  |  |
| Ulceration |  |  |  | -0.874 | 0.382 |
| Absent | 3 | 29 | 6 |  |  |
| Present | 0 | 22 | 5 |  |  |
| Surgical margins |  |  |  | 0.079 | 0.224 |
| Negative | 3 | 49 | 9 |  |  |
| Positive | 0 | 2 | 2 |  |  |
| Tumor thickness |  |  |  | -3.577 | **＜0.001**** |
| ≤4 mm | 3 | 32 | 1 |  |  |
| ＞4mm | 0 | 19 | 10 |  |  |
| Clark level |  |  |  | -5.510 | **＜0.001**** |
| Ⅰ-Ⅲ | 3 | 44 | 0 |  |  |
| Ⅳ-Ⅴ | 0 | 7 | 11 |  |  |
| Lymph node metastasis |  |  |  | -3.573 | **＜0.001**** |
| Absent | 3 | 25 | 0 |  |  |
| Present | 0 | 26 | 11 |  |  |
| AJCC stage |  |  |  |  |  |
| I / II | 2 | 24 | 0 | -2.956 | **0.003**** |
| III /I V | 1 | 27 | 11 |  |  |
| Note: ***P*＜0.01 | | | | | |

| Table S5 Cox Regression Results of Factors Influencing Survival in Cutaneous Melanoma | | | | |
| --- | --- | --- | --- | --- |
| Independent Variable | Univariate Cox  HR (95% CI) | *P* | Multivariate Cox  HR (95% CI) | *P* |
| Age |  |  |  |  |
| ＜60 | 1 |  | 1 |  |
| ≥60 | 3.232（1.379 -7.576） | **0.007**** | 1.798(0.563-5.741) | 0.322 |
| Gender |  |  |  |  |
| Male | 1 |  | 1 |  |
| Famale | 0.809(0.394-1.659) | 0.526 | 0.740(0.238-2.304) | 0.604 |
| Ulceration |  |  |  |  |
| Absent | 1 |  | 1 |  |
| Present | 0.779(0.18-11.00) | 0.510 | 1.113(0.322-3.849) | 0.865 |
| Surgical margins |  |  |  |  |
| Absent | 1 |  | 1 |  |
| Present | 0.578(0.425-4.627) | 0.578 | 0.516(0.116-2.286) | 0.384 |
| Tumor thickness |  |  |  |  |
| ≤4 mm | 1 |  | 1 |  |
| ＞4mm | 2.178(1.019-4.655) | **0.045*** | 1.941(0.689-5.464) | 0.209 |
| Clark level |  |  |  |  |
| Ⅰ-Ⅲ | 1 |  | 1 |  |
| Ⅳ-Ⅴ | 7.179(3.192-16.146) | **＜0.001**** | 1.972(0.491-7.927) | 0.339 |
| Lymph node metastasis |  |  |  |  |
| Absent | 1 |  | 1 |  |
| Present | 22.305(3.037-163.822) | **0.002**** |  | 0.917 |
| CD68^+^TAMs |  |  |  |  |
| High | 1 |  | 1 |  |
| Low | 0.241(0.107-0.546) | **＜0.001**** | 3.639(0.721-18.359) | 0.118 |
| Negative | 0.836(0.1112-6.229) | 0.861 |  | 0.829 |
| CD163^+^TAMs |  |  |  |  |
| High | 1 |  | 1 |  |
| Low | 0.105(0.049 -0.225 ) | **＜0.001**** | 0.143(0.029-0.714) | **0.018*** |
| Negative | 0.360(0.048-2.719) | 0.322 |  |  |
| AJCC stage |  |  |  |  |
| I / II | 1 |  | 1 |  |
| III /I V | 46.618(2.268-958.241) | **0.013*** |  | 0.831 |
| Note: **P*＜0.05, ***P*＜0.01. | | | | |

| Table S6 The H-score data of CD68 ^+^TAMs and CD163^+^TAMs in melanoma tumor tissues and paracancerous tissues | | |
| --- | --- | --- |
| Group | Characteristics | H-scores *(M(P25∼P75)* |
| Melanoma tumor tissues | CD68^+^TAMs | 3 (2.5∼3) |
|  | CD163^+^TAMs | 2 (1∼2.125) |
| Adjacent tissues of melanoma | CD68^+^TAMs | 1 (0∼2) |
|  | CD163^+^ TAMs | 0 (0∼1) |

| Table S7 Key upregulated DEGs in M2-type TAMs from melanoma scRNA-seq profiles | |
| --- | --- |
| DEGs | *P* |
| HIF-1α | ＜0.05 |
| PKM | ＜0.05 |
| HK2 | ＜0.05 |
| PFKB3 | ＜0.05 |
| PSAT1 | ＞0.05 |
| PSPH | ＞0.05 |
| Note: Genes highlighted are those subjected to subsequent experimental validation. | |

| 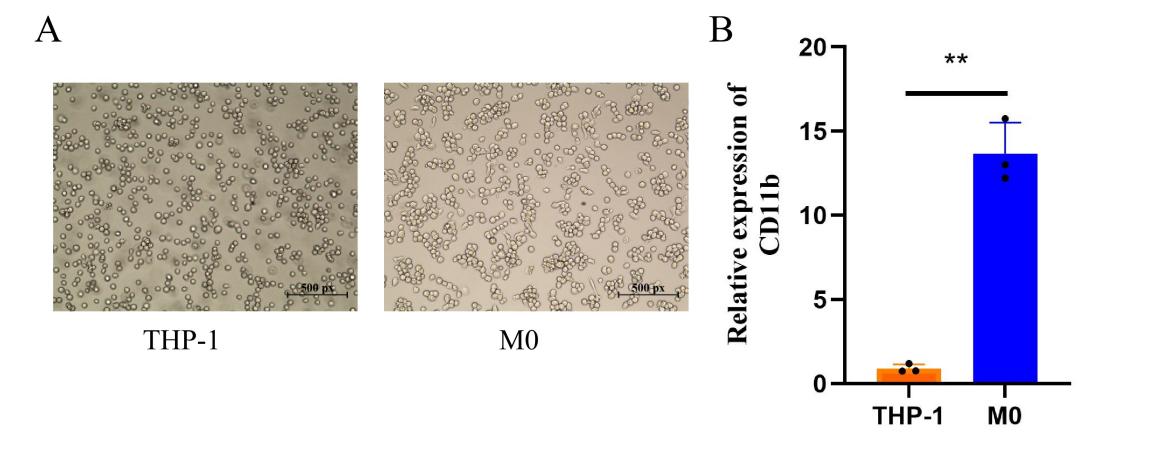 |
| --- |
| **Fig. S1** THP-1 cells were successfully differentiated into M0 macrophages.A: Morphological observation of THP-1 cells and M0 macrophages under microscopy; B: The CD11b mRNA expression was significantly upregulated in M0 macrophages compared with THP-1 cells (n=3 per group, error bars represent SD, and statistical analysis was performed using one-way ANOVA). ***P*＜0.01. |

| 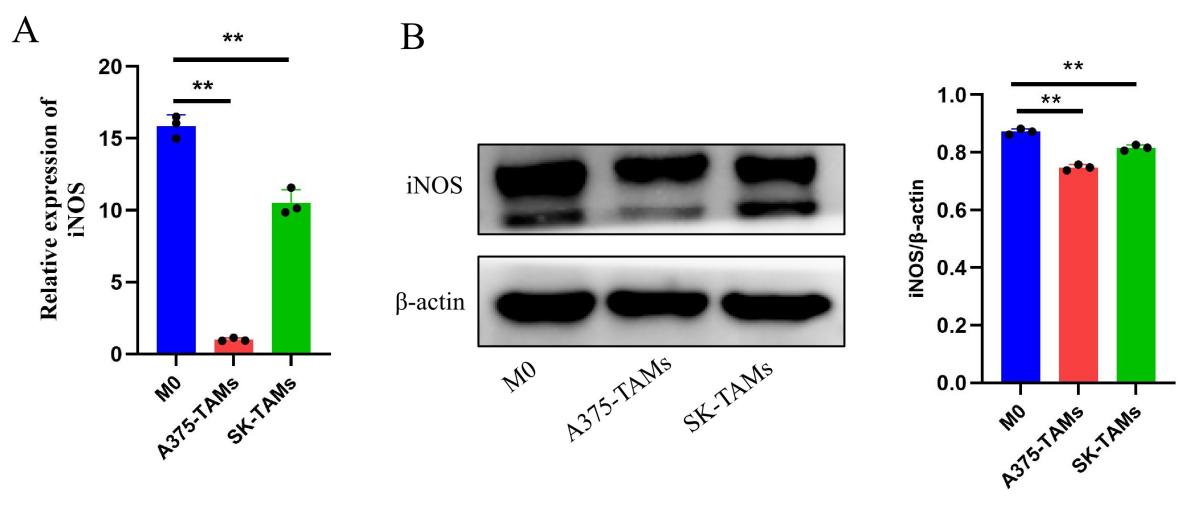 |
| --- |
| Fig. S2 The mRNA and protein expression of the M1 macrophage marker iNOS was downregulated in TAMs induced by melanoma cell culture supernatant. A: The mRNA expression of iNOS was downregulated in both A375‑TAMs and SK‑TAMs; B: The protein expression of iNOS was downregulated in both A375‑TAMs and SK‑TAMs. (n=3 per group, error bars represent SD, and statistical analysis was performed using one-way ANOVA), ***P*＜0.01. |

| 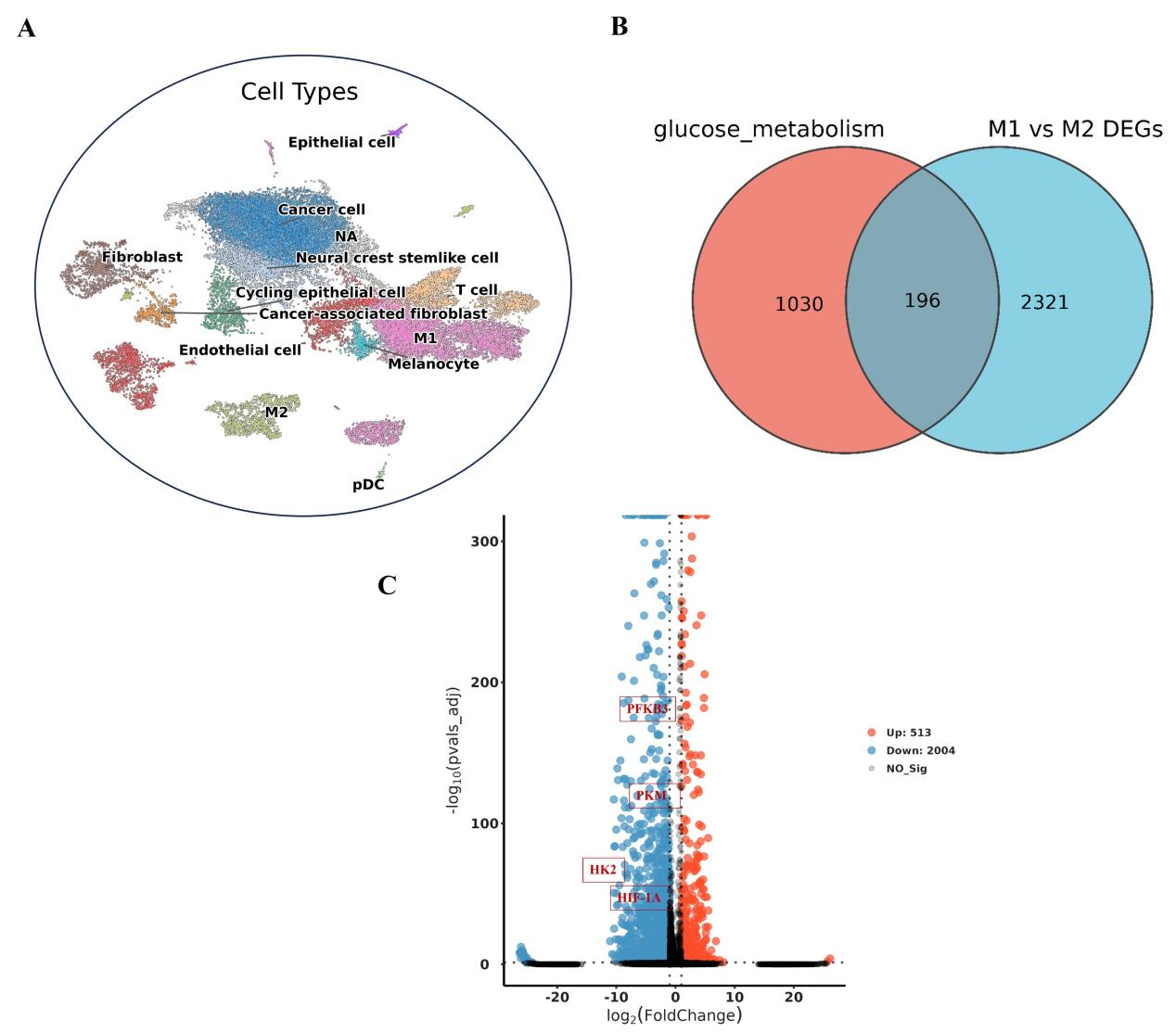 |
| --- |
| Fig. S3 Screening for key genes regulating TAMs polarization in melanoma A: Cell clustering analysis of melanoma scRNA-seq datasets; B: Schematic diagram of the intersection between DEGs of M1/M2 TAMs and glucose metabolism-related signature gene sets from scRNA-seq datasets; C: Volcano plot showing the distribution of HIF-1α and other genes among DEGs of M1/M2 TAMs. |

| 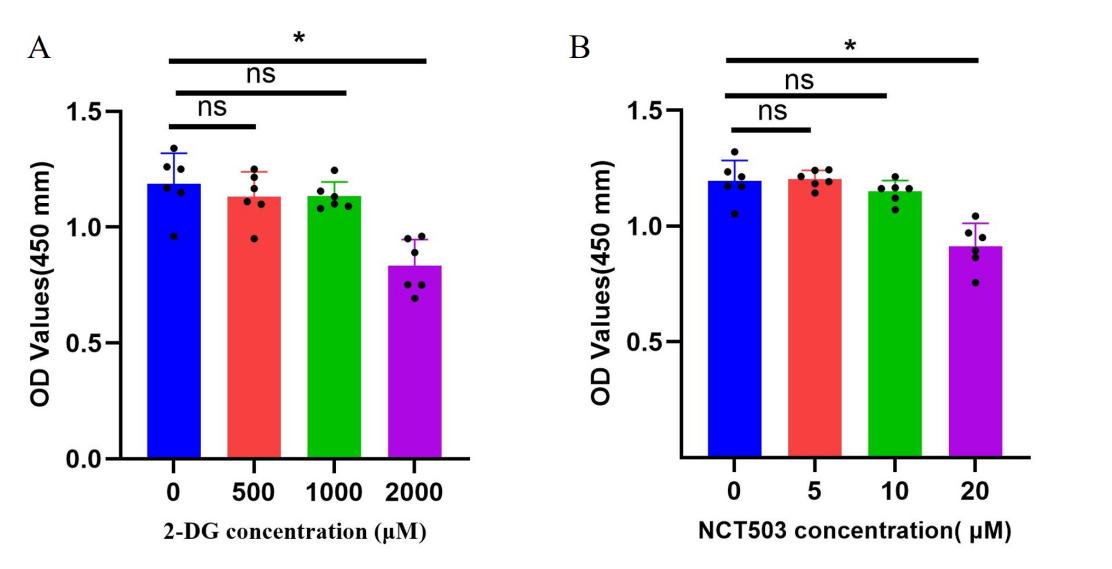 |
| --- |
| Fig. S4 Effects of 2-DG and NCT503 on TAMs viabilityA: Effect of different concentrations of 2‑DG on TAMs viability; B: Effect of different concentrations of NCT503 on TAMs viability. (n=6 per group, error bars represent SD, and statistical analysis was performed using one-way ANOVA), **P*＜0.05. |
